# Naturopathic Care for Anxiety: A Randomized Controlled Trial ISRCTN78958974

**DOI:** 10.1371/journal.pone.0006628

**Published:** 2009-08-31

**Authors:** Kieran Cooley, Orest Szczurko, Dan Perri, Edward J. Mills, Bob Bernhardt, Qi Zhou, Dugald Seely

**Affiliations:** 1 Department of Research and Clinical Epidemiology, The Canadian College of Naturopathic Medicine, Toronto, Canada; 2 Department of Social and Administrative Pharmacy, University of Toronto, Toronto, Canada; 3 Department of Clinical Pharmacology and Toxicology, University of Toronto, Toronto, Canada; 4 Department of Clinical Epidemiology & Biostatistics, McMaster University, Hamilton, Canada; 5 Institute of Medical Sciences, University of Toronto, Toronto, Canada; University of Toronto, Canada

## Abstract

**Background:**

Anxiety is a serious personal health condition and represents a substantial burden to overall quality of life. Additionally anxiety disorders represent a significant cost to the health care system as well as employers through benefits coverage and days missed due to incapacity. This study sought to explore the effectiveness of naturopathic care on anxiety symptoms using a randomized trial.

**Methods:**

Employees with moderate to severe anxiety of longer than 6 weeks duration were randomized based on age and gender to receive naturopathic care (NC) (n = 41) or standardized psychotherapy intervention (PT) (n = 40) over a period of 12 weeks. Blinding of investigators and participants during randomization and allocation was maintained. Participants in the NC group received dietary counseling, deep breathing relaxation techniques, a standard multi-vitamin, and the herbal medicine, ashwagandha (*Withania somnifera*) (300 mg b.i.d. standardized to 1.5% withanolides, prepared from root). The PT intervention received psychotherapy, and matched deep breathing relaxation techniques, and placebo. The primary outcome measure was the Beck Anxiety Inventory (BAI) and secondary outcome measures included the Short Form 36 (SF-36), Fatigue Symptom Inventory (FSI), and Measure Yourself Medical Outcomes Profile (MY-MOP) to measure anxiety, mental health, and quality of life respectively. Participants were blinded to the placebo-controlled intervention.

**Results:**

Seventy-five participants (93%) were followed for 8 or more weeks on the trial. Final BAI scores decreased by 56.5% (p<0.0001) in the NC group and 30.5% (p<0.0001) in the PT group. BAI group scores were significantly decreased in the NC group compared to PT group (p = 0.003). Significant differences between groups were also observed in mental health, concentration, fatigue, social functioning, vitality, and overall quality of life with the NC group exhibiting greater clinical benefit. No serious adverse reactions were observed in either group.

**Relevance:**

Many patients seek alternatives and/or complementary care to conventional anxiety treatments. To date, no study has evaluated the potential of a naturopathic treatment protocol to effectively treat anxiety. Knowledge of the efficacy, safety or risk of natural health products, and naturopathic treatments is important for physicians and the public in order to make informed decisions.

**Interpretation:**

Both NC and PT led to significant improvements in patients' anxiety. Group comparison demonstrated a significant decrease in anxiety levels in the NC group over the PT group. Significant improvements in secondary quality of life measures were also observed in the NC group as compared to PT. The whole system of naturopathic care for anxiety needs to be investigated further including a closer examination of the individual components within the context of their additive effect.

**Trial Registration:**

Controlled-Trials.com ISRCTN78958974

## Introduction

Anxiety disorders are among the most prevalent type of psychiatric disorder with an estimated lifetime incidence ranging between 7.9% and 14.5% worldwide [1], [2], [3]. These disorders are associated with considerable chronicity, morbidity, and disability [3], [4], [5]. Anxiety has been associated with increased risk for other disease conditions and plays an important role in overall quality of life, as well as ability to function in normal daily living. In addition, anxiety disorders impose high individual and social burden, tend to be chronic, and can be as disabling as somatic disorders. Compared with those who have other psychiatric disorders, people with anxiety disorders are high care utilizers who present to general practitioners more frequently than to psychiatric professionals, placing a strain upon the health care system and/or private insurance drug benefit plans. The economic costs of anxiety disorders include psychiatric, and nonpsychiatric emergency care; hospitalization; prescription drugs; reduced productivity; absenteeism from work; and suicide [2].

Several studies have demonstrated the economic repercussions of widespread anxiety in the workplace[2], [3], [6]. A data set from a large employer database has demonstrated both the medical and productivity costs of anxiety disorder [6]. This study concluded that, employees diagnosed with anxiety disorders were significantly more likely to have additional diagnoses, use more services, require hospitalization, or visit the emergency room compared with the control group based on analysis of work and medical records. In the final analysis, after controlling for differences in comorbidities employees diagnosed with anxiety disorders had significantly higher medical costs, productivity costs, and total costs compared with the control group [6]. Data from the World Health Organization Collaborative Study on Psychological Problems in General Health (WHO-PPGHC) reveals the impact of psychiatric illnesses and comorbidities in terms of increased disability in a primary care population [7]. This study reported the mean number of work loss days with marked disability (during the past 30 days) due to **any** anxiety disorder to be 19; higher than diabetes and heart disease.

Although complementary and alternative medicine (CAM) treatments are popular in the general population [7] as well as in those suffering from anxiety [8], [9], [10], there is limited or sometimes contradictory evidence to support their use in the treatment of anxiety disorders [8]. Previous CAM research on anxiety has generally been focused on the effectiveness of a single intervention or therapy [11], [12], with no previous studies investigating naturopathic approaches. At the same time, recent trends in the treatment of anxiety reveal a significant superiority of combination psychotherapy and pharmacological intervention over monotherapy (either psychotherapy or pharmacological treatment) [13]. This movement towards the use of multiple types of treatment for anxiety in clinical practice not only reflects the overall values and practice inherent to many CAM therapies [14], [15], but also presents many challenges to research using the randomized trial design [16], [17]. To address these concerns, CAM research often employs a pragmatic randomized controlled trial design; which is most useful to investigate whether an intervention works under real-life conditions, as well as whether it is effective for outcomes that matter to the patient [18].

Naturopathic medicine (also known as naturopathy) is a school of medical philosophy and practice that seeks to improve health and treat disease chiefly by assisting the body's innate capacity to recover from illness and injury. This alternative medical system of care employs the use of many CAM therapies including acupuncture, herbal medicine, osteopathy, nutrition, homeopathy, and lifestyle counseling[19] in a combined manner to address the underlying cause of disease. No previous studies have investigated the impact of a naturopathic approach for anxiety.

Behavioral interventions such as diaphragmatic breathing have been shown to reduce anxiety levels and improve coping capability in stressful situations [20]. Therapies identifying negative stress coping patterns and replacing them with positive outlets are effective means of controlling and treating anxiety [21]. The results of a large meta-analysis conducted by Faragher *et al* indicates that stress reduction counseling is an effective primary care treatment for widespread health concerns including mental health, anxiety, self-esteem, physical health, and job satisfaction in the workplace [22].

*Withania somnifera*, has been an important herb in use within the Ayurvedic and indigenous medical systems for over 3000 years. Clinical trials and animal research support the use of *Withania somnifera* for anxiety, inflammation, Parkinson's disease, cognitive and neurological disorders and as a useful adjunct for patients undergoing radiation and chemotherapy. *Withania somnifera* is also used therapeutically as an adaptogen for patients with nervous exhaustion, insomnia, debility due to stress, and as an immune stimulant in patients with low white blood cell counts [23], [24].

The major biochemical constituents of *Withania somnifera* root are steroidal alkaloids and steroidal lactones in a class of constituents called withanolides [23], [24]. At present, 12 alkaloids, 35 withanolides, and several sitoindosides from this plant have been isolated and studied. A sitoindoside is a withanolide containing a glucose molecule at carbon 27. Much of *Withania*'s pharmacological activity has been attributed to two main withanolides, withaferin A and withanolide D [23], [24].

Theoretically, the withanolides serve as important hormone precursors which may be convertible into human physiologic hormones as needed. *Withania* is thought to be amphoteric [23], [24]; i.e. it can help regulate important physiologic processes. The theory is that when there is an excess of a certain hormone, the plant-based hormone precursor occupies cell membrane receptor sites so the actual hormone cannot attach and exert its effect. If the hormone level is low, the plant-based hormone exerts a small effect. *Withania* may also facilitate the ability to withstand stressors, and has antioxidant properties as well. Other studies have shown *Withania somnifera* to have an immunostimulatory effect [25].

Most studies on *Withania*'s anxiolytic effect are based on animal studies. In one study the anxiolytic and antidepressive actions of *Withania somnifera* were compared to commonly prescribed pharmaceuticals. In this preclinical work, an extract of the root was administered orally to rats once daily for five days and results were compared to a group administered the anxiolytic benzodiazepine lorazepam and the tricyclic antidepressant imipramine [26]. Both the *Withania* group and the lorazepam group demonstrated reduced brain levels of a marker for clinical anxiety. Other preclinical studies confirm these results, lending support to the use of *Withania* as an antistress adaptogen [25], [27], [28], [29]. In a recently published study we demonstrated the anxiolytic potential of a compound natural health product which had *Withania* as the main herb in an open label human trial [30].

A study showing the role of a *Withania* extract on central nervous system calcium antagonism, sheds some light on a potential anxiolytic mechanism of action. In this study by Grunze *et al*, *Withania* extract supplementation resulted in extracellular calcium antagonism in neurons thereby counteracting excitation [31]. Calcium excitation has been shown to play a role in various psychiatric conditions including anxiety, and it is reasonable to expect an anxiolytic action when calcium excitation in inhibited.

Naturopathic treatment generally includes suggested alterations in patients' diet. Diets high in stimulants have been linked to symptoms of anxiety. Reducing stimulants such as caffeine, chocolate, nicotine, refined sugars will reduce incidence and severity of anxiety [32]. Studies have shown that reduction in dietary fat and alcohol also reduces the vulnerability to stress responses [33], [34].

### Objective

We aimed to determine the effectiveness of naturopathic interventions on anxiety symptoms and general health as compared to a standardized psychotherapy intervention, utilizing a randomized controlled pragmatic trial based on whole systems research trial design [35].

## Methods

### Ethics Statement

This research was conducted according to the principles expressed in the Declaration of Helsinki and received approval and oversight from the research ethics board of The Canadian College of Naturopathic Medicine and Health Canada's Natural Health Products Directorate Clinical Trial Division. Study products were manufactured and dispensed to participants under Good Manufacturing Practices and met Health Canada criteria for quality[36]. All participants provided informed consent prior to taking part in the study.

The protocol for this trial and supporting CONSORT checklist are available as supporting information; see Checklist S1 and Protocol S1.

For this study a team of naturopathic doctors at the Canadian College of Naturopathic Medicine and its associated teaching clinic (the Robert Schad Naturopathic Clinic) in Toronto, Canada provided expert advice in designing a typical (but not necessarily superlative) naturopathic intervention for anxiety symptoms. The psychotherapy intervention was chosen based on previous studies demonstrating its effectiveness[37], as well as the similarities in patient-practitioner relationship and patient education to a naturopathic intervention.

The study took place from March to July 2006 at two locations of the Canada Post Corporation (CPC) in Ontario, Canada. Canada Post employees who are members of the Canadian Union of Postal Workers (CUPW) were recruited through poster advertising. Interested employees received an information package that included a sample informed consent form, background information explaining the purpose of the study, a description of the intended care as per study protocol, a question and answer sheet regarding study participation, and contact information for study enrolment. Participants came from a range of occupations including mail carriers, transport drivers, and mail processors. Baseline characteristics of the participants are discussed below and summarized in Table 1.

**Table 1:** Baseline participant characteristics.

Parameter	Naturopathic Care (NC)	Psychotherapy (PT)	P value
Gender	26 Female∶15 Male	25 Female∶15 Male	0.93
Age	52.02±10.95	51.28±8.18	0.73
BMI	28.25±3.70	29.02±5.08	0.43
Number of people using anxiety medication	6	7	0.73
BAI score	23.54±10.13	23.45±11.82	0.97
Number of current smokers	11	8	0.24
Caffeine doses/wk*	11.78	15.91	0.209

* Caffeine doses were calculated based on reported consumption level of caffeinated drinks; 7 oz.Coffee = 1.0; 1 serving cola = 1, tea = 0.5.

Values are shown±Std Deviation.

All potential study participants were required to provide written informed consent and to undergo a 1-hour assessment with a naturopathic doctor (ND) including a non-structured psychiatric interview according to DSM-IV criteria. Self-assessed anxiety levels were quantified using the Beck Anxiety Inventory (BAI) following this interview, and participants were centrally randomized to treatment or control group following collection of this data. Participants' BAI score was utilized to screen for those who exhibited ‘moderate’ or ‘greater’ anxiety. In addition, the Beck Depression Inventory (BDI) was used to screen participants for conjoint depression as per exclusion criteria [38]. These self-report questionnaires were selected as outcome measures to remain consistent with the pragmatic nature of the study's design. All baseline outcome measures were collected at this time including secondary outcome questionnaires, seated blood pressure, and weight. Participants were given a dietary log to fill out that included details on food, alcohol, caffeine, and cigarette consumption as well as medication use. No participants were currently undergoing adjunctive psychotherapy or other counseling for the treatment of their anxiety. Data on absenteeism and expenditures on the employee health benefits plan were obtained from the employer from participants who signed an additional informed consent for the researchers to access this information for a cost-benefit analysis as done in previous studies in this occupational setting[39].

Participants were excluded if they could not comply with the study protocol, had mild or no anxiety at the time of assessment (BAI score<10), suffered from concomitant mild to severe depression (BDI score>10), were currently taking prescription medication that required **daily** doses of benzodiazepine class drugs, had previously identified allergies or sensitivity to withanolides or *Withania somnifera*, abused substances such as alcohol or illegal drugs, or had a severe concurrent illness. Participants were also excluded if they were pregnant or breastfeeding. Use of periodic or *ad lib* anxiety medications was monitored, but was not a reason for exclusion.

Two licensed naturopathic doctors conducted enrollment and provided on site delivery of care (OS, KC). This study was a randomized pragmatic control trial comparing naturopathic care to standard first-line treatment for anxiety (psychotherapy). The treatment interventions were planned for 12 weeks. After participants were considered eligible and baseline information collected, they were randomized using age and gender matched stratification to either naturopathic care (active group) (NC) or psychotherapy (control group)(PT) from one of the two practitioners (OS, KC) by an independent third party (DS) using a computer generated randomization sequence. Assignment to practitioner was based on chronology and by alternating back and forth between the practitioners at the time of enrollment (i.e. prior to any mutual knowledge or relationship of the patient and practitioner). Allocation concealment using central randomization was preserved up to the point of treatment. Although the analysts and participants were blinded to treatment allocation, and supplements were delivered using opaque, matched pills for all the supplements and placebo used, it was not possible to mask the interventions from the participants or the clinicians delivering care. Care was taken in the informed consent to ensure that participant blinding was preserved and thus keep expectation biases as equal as possible in both treatment groups.

### Treatment Groups

#### Naturopathic care (NC)

Participants receiving naturopathic care were seen once per week for 30 minutes to receive specific treatment for anxiety over a period of 12 weeks. A licensed naturopathic doctor with more than 4 years of experience administered the treatments during these sessions. During the treatment sessions, data on patients' lifestyle and diet was obtained, and diaphragmatic deep breathing exercises were performed. Lifestyle and nutritional counseling specific to the individual patient was given with special emphasis on reducing intake of stimulants (caffeine, cigarettes, chocolate), eating small, meals at regular intervals, and increasing consumption of fruit, fish, vegetables, nuts, and whole grains. Participants were encouraged to exercise on a regular basis. Two supplements were provided for participants to take throughout the trial: 1) the herb *Withania somnifera* (Swiss - Ashwagandha) (standardized to 1.5% withanolides) taken at 300 mg, two times each day, and 2) an adult multivitamin (Swiss - Senior Multi One) containing generic vitamin and mineral components approved by the Natural Health Products Directorate as a factor in the maintenance of good health. Supplements were administered to the participants, and compliance was assessed through pill count in 30-day increments for the duration of the trial. All supplements and corresponding placebo pills were manufactured and provided by Swiss Herbals using certified Good Manufacturing Practices.

Although individualized treatment of patients is a highly emphasized tenet that governs the practice of naturopathic medicine, this ‘generalized’ treatment protocol was designed by a group of experienced naturopathic doctors at a naturopathic educational institution for the purposes of this trial. Consideration was given to the overall concerns and typical needs of this population of participants in drafting the naturopathic treatment protocol, including the need to address generalized anxiety, physical and mental strain, improper sleeping schedules, a holistic view of health, overuse of stimulating substances, and support optimal health within the context of the workplace. Characteristics of the Canada Post population as well as the experiences of two naturopathic doctors (OS, KC) involved in a previous trial provided the basis for the study intervention [40]. Overall, treatments for the naturopathic group contained an element of individualized counseling, therapeutic doctor-patient relationship, patient education, a mind-body exercise, patient motivation, and a ‘pill’. Treatments involving individualized recommendations were made at the discretion of the investigator providing study care.

#### Psychotherapy (PT)

Participants randomized to this control group received psychotherapy consisting of patient directed counseling and cognitive behavioural therapy for 30 minutes each week for 12 weeks for anxiety as well as matched placebo pills. During these sessions, participants received cognitive restructuring exercises for the symptoms of anxiety delivered by a practitioner with 5 years of training and experience in cognitive behavioural therapy. In addition, participants were educated on the importance of maintaining a healthy diet, reducing stimulants (caffeine and tobacco), and taught a deep breathing exercise at the start of the trial. In an effort to provide effective control measures, participants in this group were asked about their dietary habits and stimulant use throughout the trial with no continued dietary or lifestyle counseling. Essentially, this meant that participants were made aware of the importance of these issues without ongoing advice. Participants were encouraged to exercise. Opaque, matched placebo pills (2 pills, taken twice daily) consisting of an inert fiber substance were administered and participant compliance was assessed identically to that of the naturopathic group. Careful consideration was given in the design of the treatments to provide an effective treatment for anxiety while creating an adequately matched ‘placebo’ to the active group As such, the control group treatments contained an element of individualized counseling, therapeutic doctor-patient relationship, patient education, a mind-body exercise, patient motivation and a ‘pill’. Treatments involving individualized recommendations were made at the discretion of the investigator providing study care.

### Outcomes

#### Primary Outcome

The primary outcome for this study was the *Beck Anxiety Inventory* (BAI). This questionnaire is designed to measure subjective symptoms of anxiety, as well as discriminate between anxiety and depression. The BAI consists of 21 items, each describing a common symptom of anxiety. The items are summed to obtain a total score that can range from 0–63. The scale has high internal consistency and validity [41].

### Secondary Outcomes

Secondary outcome measures included three questionnaires related to quality of life administered at 4, 8 and 12-week time points:

1. *The short form –36* (SF-36) questionnaire is a self-administered, 36-item questionnaire that measures health-related quality of life in eight domains: physical functioning, limitations due to physical problems, limitations due to emotional problems, vitality, measuring perceived level of energy and fatigue, freedom from bodily pain, social functioning, mental health, emotional state, and general health perceptions. Both physical and mental summary scores can be obtained. Each domain is scored separately from 0 (worst score) to 100 (best score). Two summary scores can be calculated from information obtained in the 8 domains- Physical Function and Mental Health Summary Scores [42].
2. *The Fatigue Questionnaire* (FQ) is a 21-item instrument developed for use in population studies to measure physical and mental aspects of fatigue, a feature commonly observed in many somatizing patients with anxiety. Each item has 7 response options, with lower scores corresponding to high levels of fatigue [43].
3. *Measure Yourself Medical Outcomes Profile* (MYMOP) is a patient-centered outcome questionnaire with internal consistency and construct validity [44], [45], [46]. It has been used extensively in primary care and complementary medicine settings as a means of garnering and quantifying qualitative patient experiences. Patients choose two personally relevant symptoms of greatest importance to their individual health and rate these symptoms on a 7-point visual analogue scale. Higher scores correspond to a lower satisfaction (i.e. worse) level of health as it pertains to individually selected symptoms.

In addition, a series of 7-point visual analogue scale (VAS) questions were administered at 4, 8, and 12-week time points to measure participants subjective impressions of their own: compliance to the treatment protocol; benefit from the treatment; how well the study treatments met their expectations for treating their anxiety; interest in pursuing treatments; and ability to cope with stress since starting study treatments. Higher scores on the VAS corresponded to greater positive subjective impression in the individual parameter. Although the study was not powered to measure significance in these VAS measures, it was expected that group comparison would allow inference on the nature and variance in the non-specific effects between active and control group. We hypothesized that minimal inter-group differences in these parameters would correspond to a high correlation in non-specific effects between the two groups; essentially well-matched comparator treatments [47].

### Statistical analysis

All analyses were performed by a statistician (QZ) under blinded conditions using SAS/STAT (Version 9.1, Cary, NC). The data at baseline visit and week 12 visit were summarized by mean and standard deviation. When the missing data occurred at week 12, its value was replaced by the value at week 8 as part of intention to treat analysis. For each group, the treatment effect at week 12 was compared to the baseline and assessed by the paired t-test. The mean difference, its 95% confidence interval and p-value were reported. The difference of the treatment effect between the NC group and the PT group was compared by the independent t-test. The mean difference of the treatment effect, its 95% confidence interval and p-value are reported. The decision to not conduct a sensitivity analysis between per-protocol and intention to treat was made prior to the conduct of the study and then re-evaluated post hoc. This decision was based on the low number of dropouts prior to week 8 and minimal adverse reactions expected.

### Sample size

In order to determine a 20% reduction in anxiety scores, and assuming a population standard deviation of 20%, we calculated a sample size of 37 was necessary in each arm, providing 80% power at a 5% alpha value [48]. To supplement expected loss-to-follow-up, we were committed to enrolling at least 80 participants (at least 40 in each arm).

All patients with available data were analyzed according to the arm to which they were randomized using intention-to-treat. We summarized the baseline data and the data at week 12 by mean and standard deviation. For any missing data at week 12, we carried forward the value at week 8. The means over the 12-week period were plotted for the outcomes of BAI, SF-36, and FQ component separately. To assess the treatment effect for each group we calculated the mean change in scores between groups at week 12 and the baseline. We also calculated the mean changes between groups to examine the group effect. The statistical significance of the changes for each group were tested using the paired t-test; the exact 2-sided p-value is reported. To compare the change scores between groups, the two-sample t-test was performed. All p-values are 2-sided. Statistical analyses were done with the SAS (version 9.1) software package.

Outcomes reported from these analyses include: FQ dimension score, weight and BMI (Table 2); BAI and perceived benefit questionnaire (Table 3); SF-36 (Table 4) and adverse reactions (Table 5).

**Table 2:** Fatigue questionnaire, Weight and BMI results.

Outcomes	Group	Baseline Mean±Std (N)	Week 12^*^ Mean±Std (N)	Change at 12 wks from baseline Mean (95%CI) (N, p-value)	Difference of changes between groups Mean (95%CI) P-value
FQ Subjective	NC	66.55±17.96 (41)	47.17±20.52 (36)	−20.39 (−26.41, −14.37) (36, <0.0001)	−18.01(−25.16, −10.85) <0.0001
	PT	65.98±15.79 (40)	63.23±15.01 (39)	−2.38 (−6.63, 1.87) (39, 0.2634)	
FQ physical	NC	52.03±21.96 (41)	38.89±18.69 (36)	−14.29 (−20.63, −7.95) (36, <0.0001)	−13.19 (−21.85, −4.52) 0.0033
	PT	55.12±23.08 (40)	53.60±17.23 (39)	−1.10 (−7.22, 5.02) (39, 0.7182)	
FQ Motivation	NC	56.79±19.82 (41)	39.48±16.49 (36)	−18.95 (−26.30, −11.60) (36, <0.0001)	−20.32 (−29.05, −11.59) <0.0001
	PT	52.23±17.09 (40)	53.57±13.56 (39)	1.37 (−3.81, 6.56) (39, 0.5946)	
FQ Concentration	NC	61.74±17.37 (41)	45.24±19.34 (36)	−17.14 (−23.75, −10.54) (36, <0.0001)	−17.51 (−25.89, −9.13) <0.0001
	PT	56.00±17.93 (40)	55.82±20.44 (39)	0.37 (−5.10, 5.83) (39, 0.8928)	
Weight	NC	76.60±13.16 (41)	74.76±12.22 (36)	−1.98 (−2.85, −1.11) (36, <0.0001)	−1.47 (−2.64, −0.30) 0.0146
	PT	81.13±18.96 (40)	80.42±19.06 (39)	−0.51 (−1.32, 0.30) (39, 0.2089)	
BMI	NC	28.25±3.70 (41)	27.53±3.14 (36)	−0.75 (−1.08, −0.42) (36, <0.0001)	−0.56 (−1.00, −0.12) 0.0128
	PT	29.02±5.08 (40)	28.80±5.08 (39)	−0.19 (−0.49, −0.11) (39, 0.2136)	

Week 12^*^ - the week 8 value was used for week 12 if the week 12 data was missing.

NC = Naturopathic Care; PT = Psychotherapy.

**Table 3:** BAI, MY-MOP and Perceived Benefit questionnaire results.

Outcomes	Group	Baseline Mean±Std (N)	Week 12^*^ Mean±Std (N)	Change at 12 wks from baseline Mean (95%CI) (N, p-value)	Difference of changes between groups Mean (95%CI) P-value
BAI total score	NC	23.54±10.13 (41)	10.89±11.69 (36)	−13.31(−16.51, −10.12) (36, <0.0001)	−6.16 (−10.24, −2.08)0.0036
	PT	23.45±11.82 (40)	16.28±10.89 (39)	−7.15 (−9.84, −4.47) (39, <0.0001)	
MY-MOP Symptom 1	NC	5.20±1.25 (41)	2.88±1.25 (36)	−2.24 (−2.75, −1.72) (36, <0.0001)	−1.77 (−2.59, −0.95) <0.0001
	PT	4.43±1.45 (40)	3.97±1.53 (39)	−0.46 (−1.10, 0.17) (39, 0.1492)	
MY-MOP Symptom 2	NC	4.78±1.23 (40)	2.790±1.49 (34)	−1.94 (−2.43, −1.45) (34, <0.0001)	−1.08 (−1.90, −0.25) 0.0115
	PT	4.84±1.69 (38)	3.87±1.36 (38)	−0.86 (−1.53, −0.20) (37, 0.0126)	
Compliance (VAS measure)**	NC	4.79±1.51 (40)	5.29±0.94 (36)	0.66 (0.30, 1.01) (35, 0.0006)	0.28 (−0.16, 0.71) 0.2071
	PT	4.92±1.24 (39)	5.37±1.16 (39)	0.38 (0.11, 0.65) (38, 0.0066)	
Benefit**	NC	4.70±1.80 (40)	5.79±0.92 (36)	1.24 (0.67, 1.82) (35, 0.0001)	1.14 (0.46, 1.82) 0.0014
	PT	4.58±1.47 (40)	4.74±1.23 (39)	0.10 (−0.31, 0.51) (39, 0.6164)	
Interest **	NC	5.60±1.44 (39)	5.76±1.24 (36)	0.264 (−0.23, 0.76) (34, 0.2870)	0.008 (−0.64, 0.65) 0.9795
	PT	5.08±1.59 (40)	5.28±1.23 (39)	0.256 (−0.17, 0.69) (39, 0.2351)	
Expectation**	NC	4.80±1.59 (40)	5.57±1.09 (36)	1.01 (0.49, 1.54) (35, 0.0004)	0.73 (0.08, 1.38) 0.0272
	PT	4.55±1.32 (40)	4.77±1.17 (39)	0.28 (−0.13, 0.69) (39, 0.1722)	
Stress**	NC	5.00±1.26 (40)	5.83±0.85 (36)	0.91 (0.48, 1.35) (35, 0.0001)	0.81 (0.24, 1.37) 0.0055
	PT	4.41±1.23 (39)	4.45±1.11 (38)	0.10 (−0.27, 0.49) (37, 0.5646)	

Week 12^*^ - the week 8 value will be used for week 12 If missing value is occurred at week 12.

** - week 4 was considered baseline for these outcome measures.

NC = Naturopathic Care; PT = Psychotherapy.

**Table 4:** SF-36 results.

Outcomes SF-36	Group	Baseline Mean±Std (N)	Week 12^*^ Mean±Std (N)	Change at 12 wks from baseline Mean (95%CI) (N, p-value)	Difference of changes between groups Mean (95% CI) P-value
Aggregate physical component	NC	46.23±7.99 (36)	49.83±7.24 (34)	3.76 (1.28, 6.23) (32, 0.0042)	3.26 (−0.15, 6.66) 0.0608
	PT	45.51±8.82 (39)	46.59±7.98 (38)	0.50(−1.91, 2.91) (37, 0.6768)	
Aggregate mental component	NC	38.97±9.81 (36)	50.92±7.29 (34)	12.56 (9.02, 16.10) (32, <0.0001)	10.34 (5.21, 15.46) 0.0001
	PT	39.03±9.95 (39)	41.54±10.61 (38)	2.23 (−1.54, 5.99) (37, 0.2378)	
Physical functioning	NC	44.15±10.95 (41)	50.43±8.73 (36)	5.61 (3.10, 8.13) (36, <0.0001)	4.88 (0.99, 8.76) 0.0145
	PT	44.69±11.19 (40)	46.02±11.44 (39)	0.73 (−2.27, 3.74) (39, 0.6334)	
Role physical	NC	44.99±7.88 (39)	48.62±8.41 (36)	4.34(1.61, 7.07) (35,0.0028)	(−0.11, 7.78) 0.0564
	PT	44.18±9.49 (40)	44.86±8.92 (39)	0.50 (−2.40, 3.41) (39, 0.7181)	
Bodily pain	NC	40.55±8.63 (39)	48.04±7.93 (35)	7.77 (5.30, 10.24) (41, <0.0001)	4.40 (0.96, 7.85) 0.0130
	PT	41.07±9.66 (40)	45.06±8.41 (38)	3.36 (0.89, 5.84) (40, 0.0091)	
General health	NC	43.03±9.95 (40)	49.91±9.38 (35)	6.70 (3.74, 9.65) (35, <0.0001)	7.13 (3.34, 10.92) 0.0004
	PT	43.77±8.74 (40)	43.49±7.13 (39)	−0.43 (−2.95, 2.08) (39, 0.7286)	
Vitality	NC	42.50±9.88 (41)	53.31±10.08 (36)	11.27 (7.46,15.09) (36, <0.0001)	9.43 (4.77, 14.09) 0.0001
	PT	42.10±8.37 (40)	44.17±8.65 (39)	1.84 (−1.05, 4.73) (39, 0.2046)	
Social functioning	NC	37.90±11.32 (40)	48.12±7.85 (35)	10.27 (6.06, 14.48) (34, <0.0001)	7.40 (2.07, 12.72) 0.0072
	PT	38.95±11.19 (39)	42.31±9.64 (39)	2.87 (−0.61, 6.35) (38, 0.1031)	
Role emotional	NC	39.65±8.48 (40)	48.54±8.80 (36)	9.22 (5.40, 13.04) (35, <0.0001)	7.42 (1.86, 12.99) 0.0096
	PT	40.33±10.64 (40)	42.32±13.13 (39)	1.79 (−2.32, 5.91) (39, 0.3833)	
Mental health	NC	40.22±10.75 (40)	48.83±11.03 (36)	9.49 (5.63, 13.36) (35, <0.0001)	6.84 (1.69, 12.10) 0.0102
	PT	40.79±9.06 (40)	43.65±9.55 (39)	2.60 (−1.03, 6.23) (39, 0.1553)	

Week 12^*^ - the value at week 8 was used for week 12 If the missing value was occurred.

NC = Naturopathic Care; PT = Psychotherapy.

**Table 5:** Adverse reactions.

Type	NC	PT
Gastrointestinal upset	2	2
Overstimulation	2	2
Rash	0	1
‘Feeling warm’	1	0
Increased frequency of nocturnal night cramps	1	1
Mild hair loss	1	1

NC = Naturopathic Care; PT = Psychotherapy.

## Results

### Recruitment and Follow-up of Patients

**Figure 1:**
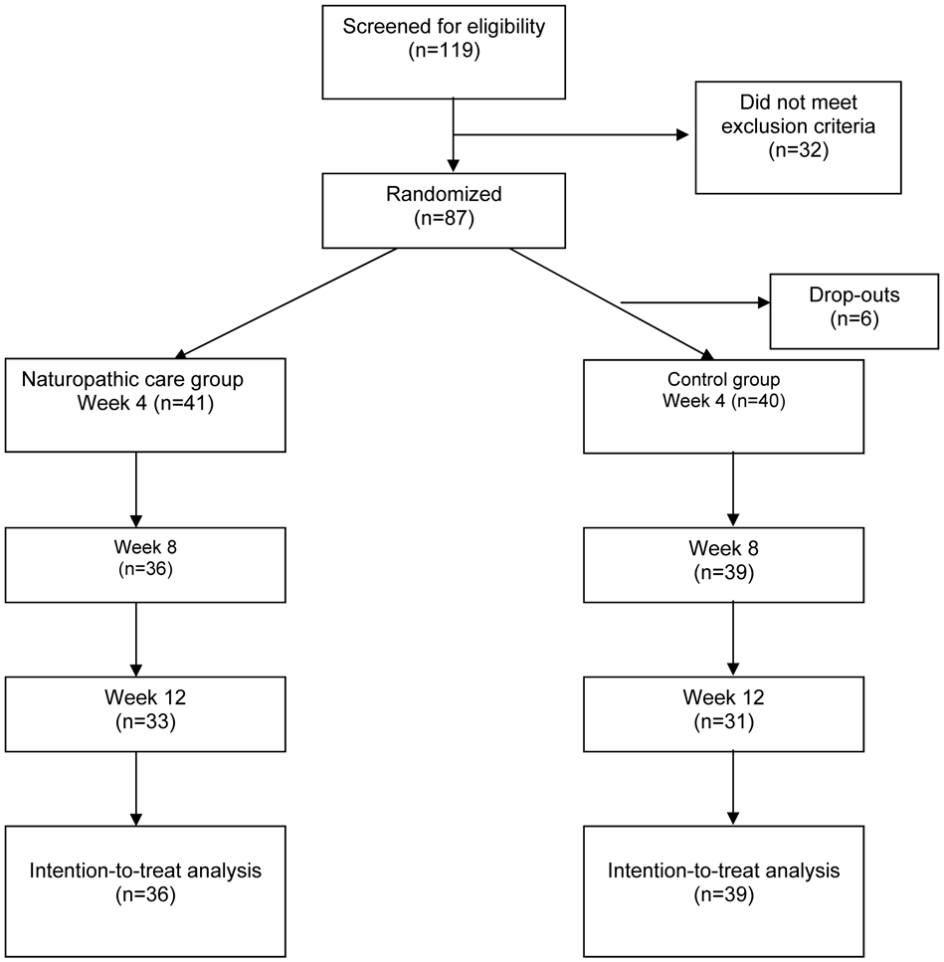
Flowchart of participants through trial.

Figure 1 describes the flow of participants through the trial. 119 people were screened, with 87 meeting inclusion/exclusion criteria. Of the 32 excluded participants, 16 had zero to mild anxiety (BAI<10), 4 had mild to severe conjoint depression (BDI>10), 2 were consuming daily doses of prescription benzodiazepine medication for treatment of their anxiety, and 10 could not commit to the time required to take part in the trial. Patients that were identified as having mild to severe depression were referred for appropriate treatment. Age and gender based central randomization occurred for the remaining 87. OS provided study care to 43 participants; KC provided study care to 44 participants. Six of the 87 participants withdrew from the study before starting treatment; three of whom had been randomized to the active treatment group and three randomized to the control group. Four of the six cited time constraints as a reason for withdrawal, and two participants had major changes in their health status and were advised not to participate in the study on the advice of their family physician. Baseline analysis was conducted with the remaining 81 participants that began treatment. During the course of the study, eight patients withdrew from the NC group, and nine withdrew from the PT group. A total of nine patients withdrew due to time constraints, six patients were lost on follow-up with no discernable reason cited for withdrawal, two patients (both from PT group) withdrew on the advice of their medical doctor and one patient was required to withdraw due to a positive pregnancy test.

### Baseline characteristics

Randomization was stratified based on age and gender. Both treatment groups had similar characteristics including baseline BAI score, number of users of prescription anxiety medication, and use of stimulants (number of smokers, use of caffeine). Table 1 provides a summary of baseline characteristics.

### Study treatments

Study treatments were well received, as shown by expectation scores demonstrated by the VAS and good compliance rates in both groups. The mean total of missed supplements was 6.83 pills (94.3%) in the NC group and 6.43 pills (94.6%) in the PT group throughout the 12-week duration of the trial.

### Outcomes

**Figure 2:**
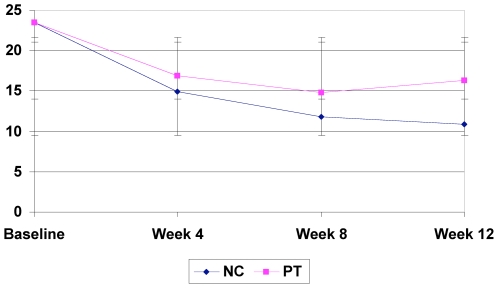
Beck Anxiety Inventory results.

**Figure 3:**
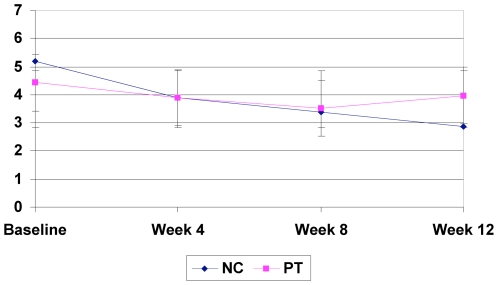
MYMOP – Symptom 1 results.

**Figure 4:**
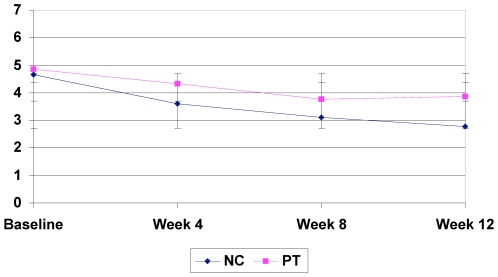
MYMOP – Symptom 2 results.

Significant improvements from baseline on the primary outcomes measure (BAI) were seen in both the naturopathic (−13.31; p-value<0.0001) and psychotherapy (−7.15; p-value<0.0001) groups (see Table 3). There was significantly greater improvement in the BAI in the naturopathic treatment group as opposed to the psychotherapy group (p = 0.003) (Fig. 2). Statistically significant differences as well as trends for differences were observed between groups in FQ subscales; subjective fatigue (p<0.0001), physical fatigue (p = 0.003), motivation (p<0.001), and concentration (p = <0.0001) with NC group showing greater improvement as compared to PT (Table 2). Patient centered outcomes as measured by the MYMOP questionnaire showed significant reductions in symptoms 1 (p<0.0001) (Fig. 3) and symptom 2 (p = 0.012) (Fig. 4) in the NC group as compared to the PT group (Table 3). Other statistically significant differences occurred in SF-36 aggregate mental component (p<0.0001), General Health (p = 0.0004), Vitality (p<0.0001), Social Functioning (p = 0.007), Role Emotional (0.0096) and Mental Health (p = 0.010) subscales as well as on mental (p<0.0001) aggregate scores (Table 4). A non-significant trend between group improvements was observed in aggregate physical component (p = 0.061), and Role Physical (p = 0.056) and Bodily Pain (p = 0.013) subscales.

### Adverse Events

Table 5 summarizes the frequency of all adverse reactions observed and/or reported according to treatment group. No differences were observed between groups; gastrointestinal upset and feeling of ‘overstimulation’ were the most commonly reported in both groups. Gastrointestinal complaints consisted of incidence of nausea or stomach pain lasting 1–2 hours after taking the pills. Complaints of ‘overstimulation’ (participant's word) include feelings of increased mental and, or physical agitation lasting 4–6 hours. All reported reactions were considered to be mild by participants reporting them.

### Costs effectiveness

22 (25%) of participants were willing to give consent for the researchers to access absenteeism and data on health care expenditures under the employee health benefits insurance program. A sample size this small proved to be unsuitable for cost-effectiveness analysis and is a limitation of the research study. This limitation based on participation may, in part, be due to increased apprehension surrounding issues of informed consent and researcher access to this information in the study's population.

## Discussion

Our results should be of interest to patients, clinicians and workplace-related health services alike. Results of this study indicate a significant reduction in anxiety level from the use of either naturopathic care or a standardized psychotherapy intervention in this workplace setting. These results are consistent with systematic reviews on the short-term efficacy of counseling in the treatment of anxiety [49]. Further research studies on the effects of either of these interventions in other workplace settings are warranted, particularly with respect to impact on worker presenteeism and cost-effectiveness based on larger-scale implementation. Comparatively, naturopathic treatment showed a clinically and statistically significant improvement in anxiety and quality of life measures as compared to the control standard.

Our study demonstrates that naturopathic treatments, cognitive behavioral therapy and counseling have a significant effect on decreasing the symptoms of anxiety. In addition, naturopathic treatments including dietary changes and the herb *Withania somnifera* have additional secondary effects on quality of life of individuals in the workplace, including significant reduction in stress and improved vitality, motivation, general health, and patient-specific concerns. Few participants in the study were receiving treatment for anxiety despite remarkable symptoms of anxiety and dysfunctional mental health. This observation is consistent with the persisting stigma surrounding mental health disorders and reticence to explore treatments [50]. The benefits from this pragmatic study on providing access to care and ongoing treatment for all the participants were notable and highly valued by the workplace.

### Limitations

This was a pragmatic randomized controlled trial, and as such, it is difficult to ascertain the precise effect of each of the component therapies of the NC and PT treatment packages. Future research trials employing a multi-factorial design would assist in isolating and characterizing the effects of single and combination treatments.

Maintaining internal and external validity in pragmatic RCTs is a challenge [51], [52]. Our study design attempted to maximize external validity (generalizability) by having few exclusion criteria and including some flexibility or variability in study interventions based on individual needs (e.g. specific advice given in counseling or dietary advice). However, one specific herbal component was used to treat all study participants in the NC group despite the range of potential psychoactive herbs with varying degrees of supportive evidence [53]. Treatment decisions made by the naturopathic doctors in the study was limited as a result, thus decreasing the external validity of our study. The overall generalizability of our results is limited, in part, due to small sample size.

Internal validity was achieved by: attempting to account for non-specific effects of treatments in both groups; absence of assessment bias; and preventing contamination [51], [52]. Both the NC and PT groups received: individualized counseling, therapeutic doctor-patient relationship, patient education, a mind-body exercise, patient motivation, and a ‘pill’. In addition, both treatment groups reported that their expectations for treatment were being effectively met on 7-point VAS throughout the course of the study (NC: 5.57, PT: 4.77). Assessment and data analysis was conducted blind to group allocation. No contamination of the PT group with respect to *Withania* use or visits to a naturopathic doctor occurred. No patients in either group were undergoing active counseling or psychotherapy outside this study. Lack of a ‘no treatment’ control group limits the internal validity of this study; we are unable to account for spontaneous changes in anxiety levels or regression to the mean within the study design. However, the natural course and severity of anxiety is generally consistent over time when it has been present with a large degree of chronicity (>6 months)[2].

Although a statistically significant difference in reduction of anxiety symptoms was observed between NC and PT treatment, we observed some methodological challenges based on our study population and study design. Sample size calculation was based on detecting a clinically and statistically significant effect size (±20%) on BAI scores from baseline, however both the NC and PT groups demonstrated significant reductions in BAI scores (NC 56.5%, PT 30.5%) thus minimizing the ability to detect the differential effect size [54]. In addition, the heterogeneity of the study participants, as reflected by the large variability in BAI scores, impacts negatively on predictability and the ability to detect between group differences [54].

Additionally, it should be highlighted that this study investigated changes in patient-reported symptoms of anxiety. Future studies would need to employ the use of blinded independent assessment of this disorder using a structured diagnostic interview based on DMS-IV criteria (SCID) throughout the study to gather non-patient rated information relating to anxiety.

### Conclusion

Naturopathic treatment including *Withania somnifera*, a multi vitamin, dietary counseling and cognitive-behavioral therapy appears to be a safe and effective, with benefit over standardized psychotherapy in the treatment of mild to severe generalized anxiety in the Canada Post worker population. Future research is required to examine effects of the individual components of treatments as well as the nature of the therapeutic relationship in mental health disorders like anxiety.

## Supporting Information

Checklist S1 CONSORT checklist
(0.05 MB DOC)

Protocol S1 Trial Protocol
(0.45 MB DOC)

## References

[1] Ustun TB, Sartorius N (1995). Mental illness in general health care: an international study..

[2] Lepine JP (2002). The epidemiology of anxiety disorders: prevalence and societal costs.. J Clin Psychiatry.

[3] Alonso J, Lepine JP (2007). Overview of Key Data From the European Study of the Epidemiology of Mental Disorders (ESEMeD).. J Clin Psychiatry.

[4] Brawman-Mintzer O, Yonkers KA (2004). New trends in the treatment of anxiety disorders.. CNS Spectr.

[5] Howell HB, Brawman-Mintzer O, Monnier J, Yonkers KA (2001). Generalized anxiety disorder in women.. Psychiatr Clin North Am.

[6] Marciniak M, Lage MJ, Landbloom RP, Dunayevich E, Bowman L (2004). Medical and productivity costs of anxiety disorders: case control study.. Depress Anxiety.

[7] Rafferty AP, McGee HB, Miller CE, Reyes M (2002). Prevalence of complementary and alternative medicine use: state-specific estimates from the 2001 Behavioral Risk Factor Surveillance System.. Am J Public Health.

[8] Brown RP, Gerbarg PL (2001). Herbs and nutrients in the treatment of depression, anxiety, insomnia, migraine, and obesity.. J Psychiatr Pract.

[9] Krisanaprakornkit T, Krisanaprakornkit W, Piyavhatkul N, Laopaiboon M (2006). Meditation therapy for anxiety disorders.. Cochrane Database Syst Rev.

[10] Roy-Byrne PP, Bystritsky A, Russo J, Craske MG, Sherbourne CD (2005). Use of herbal medicine in primary care patients with mood and anxiety disorders.. Psychosomatics.

[11] Pittler MH, Ernst E (2003). Kava extract for treating anxiety.. Cochrane Database Syst Rev.

[12] Pilkington K, Kirkwood G, Rampes H, Fisher P, Richardson J (2006). Homeopathy for anxiety and anxiety disorders: a systematic review of the research.. Homeopathy.

[13] Furukawa T, Watanabe N, Churchill R (2007). Combined psychotherapy plus antidepressants for panic disorder with or without agoraphobia.. Cochrane Database Syst Rev.

[14] Borgerson K (2005). Evidence-based alternative medicine?. Perspect Biol Med.

[15] Elder C, Aickin M, Bell IR, Fonnebo V, Lewith GT (2006). Methodological challenges in whole systems research.. J Altern Complement Med.

[16] Timmermans S, Berg M (2003). The gold standard: The challenge of evidence based medicine and standardization in health care..

[17] Verhoef MJ, Lewith G, Ritenbaugh C, Boon H, Fleishman S (2005). Complementary and alternative medicine whole systems research: beyond identification of inadequacies of the RCT.. Complement Ther Med.

[18] Hotopf M, Churchill R, Lewis G (1999). Pragmatic randomised controlled trials in psychiatry.. Br J Psychiatry.

[19] Smith MJ, Logan AC (2002). Naturopathy.. Med Clin North Am.

[20] Somer E, Tamir E, Maguen S, Litz BT (2005). Brief cognitive-behavioral phone-based intervention targeting anxiety about the threat of attack: a pilot study.. Behav Res Ther.

[21] Leahy RL (2003). Cognitive therapy techniques: a practitioner's guide..

[22] Faragher EB, Cass M, Cooper CL (2005). The relationship between job satisfaction and health: a meta-analysis.. Occup Environ Med.

[23] (2004). Monograph. Withania somnifera.. Altern Med Rev.

[24] Mishra LC, Singh BB, Dagenais S (2000). Scientific basis for the therapeutic use of Withania somnifera (ashwagandha): a review.. Altern Med Rev.

[25] Archana R, Namasivayam A (1999). Antistressor effect of Withania somnifera.. J Ethnopharmacol.

[26] Bhattacharya SK, Bhattacharya A, Sairam K, Ghosal S (2000). Anxiolytic-antidepressant activity of Withania somnifera glycowithanolides: an experimental study.. Phytomedicine.

[27] Bhattacharya SK, Muruganandam AV (2003). Adaptogenic activity of Withania somnifera: an experimental study using a rat model of chronic stress.. Pharmacol Biochem Behav.

[28] Singh B, Chandan BK, Gupta DK (2003). Adaptogenic activity of a novel withanolide-free aqueous fraction from the roots of Withania somnifera Dun. (Part II).. Phytother Res.

[29] Dhuley JN (1998). Effect of ashwagandha on lipid peroxidation in stress-induced animals.. J Ethnopharmacol.

[30] Seely D, Singh R (2007). Adaptogenic Potential of a Polyherbal Natural Health Product: Report on a Longitudinal Clinical Trial.. eCAM.

[31] Grunze H, Langosch J, von Loewenich C, Walden J (2000). Modulation of neural cell membrane conductance by the herbal anxiolytic and antiepileptic drug aswal.. Neuropsychobiology.

[32] Rainey JM, Frohman CE, Freedman RR, Pohl RB, Ettedgui E (1984). Specificity of lactate infusion as a model of anxiety.. Psychopharmacol Bull.

[33] Straznicky NE, Louis WJ, McGrade P, Howes LG (1993). The effects of dietary lipid modification on blood pressure, cardiovascular reactivity and sympathetic activity in man.. J Hypertens.

[34] Monteiro MG, Schuckit MA, Irwin M (1990). Subjective feelings of anxiety in young men after ethanol and diazepam infusions.. J Clin Psychiatry.

[35] Ritenbaugh C, Verhoef M, Fleishman S, Boon H, Leis A (2003). Whole systems research: a discipline for studying complementary and alternative medicine.. Altern Ther Health Med.

[36] Canada H (2009). Natural Health Product Regulations.

[37] Erickson DH, Janeck AS, Tallman K (2007). A cognitive-behavioral group for patients with various anxiety disorders.. Psychiatr Serv.

[38] Osman A, Kopper BA, Barrios F, Gutierrez PM, Bagge CL (2004). Reliability and validity of the Beck depression inventory—II with adolescent psychiatric inpatients.. Psychol Assess.

[39] Herman PM, Szczurko O, Cooley K, Mills EJ (2008). Cost-effectiveness of naturopathic care for chronic low back pain.. Altern Ther Health Med.

[40] Szczurko O, Cooley PK, Busse JW, Seely D, Bernhardt B (2007). Naturopathic care for chronic low back pain: A randomized controlled trial.. Public Library of Science - One *In press*.

[41] Kick SD, Bell JA, Norris JM, Steiner JF (1994). Validation of two anxiety scales in a university primary care clinic.. Psychosom Med.

[42] Ware JJE, Spilker B (1996). The SF-36 health survey.. Quality of life and pharmacoeconomics in clinical trials.

[43] Hann DM, Jacobsen PB, Azerello LM, Martin SC, Curran SL (1998). Measurement of fatigue in cancer patients: development and validation of the Fatigue Symptom Inventory.. Quality of Life Research.

[44] Paterson C, Britten N (2000). In pursuit of patient-centred outcomes: a qualitative evaluation of the ‘Measure Yourself Medical Outcome Profile’.. J Health Serv Res Policy.

[45] Paterson C, Langan CE, McKaig GA, Anderson PM, Maclaine GD (2000). Assessing patient outcomes in acute exacerbations of chronic bronchitis: the measure your medical outcome profile (MYMOP), medical outcomes study 6-item general health survey (MOS-6A) and EuroQol (EQ-5D).. Qual Life Res.

[46] Paterson C (1996). Measuring outcomes in primary care: a patient generated measure, MYMOP, compared with the SF-36 health survey.. Bmj.

[47] Jonas WB, Lewith G, Walach H, Lewith G, Jonas WB, Walach H (2002). Balanced research strategies for complementary and alternative medicine.. Clinical research in complementary therapies: principles, problems and solutions.

[48] Rosner B (2001). Fundamentals of biostatistics.

[49] Bower P, Rowland N, Hardy R (2003). The clinical effectiveness of counselling in primary care: a systematic review and meta-analysis.. Psychol Med.

[50] Nutt DJ, Kessler RC, Alonso J, Benbow A, Lecrubier Y (2007). Consensus statement on the benefit to the community of ESEMeD (European Study of the Epidemiology of Mental Disorders) survey data on depression and anxiety.. J Clin Psychiatry.

[51] Godwin M, Ruhland L, Casson I, MacDonald S, Delva D (2003). Pragmatic controlled clinical trials in primary care: the struggle between external and internal validity.. BMC Med Res Methodol.

[52] van der Windt DA, Koes BW, van Aarst M, Heemskerk MA, Bouter LM (2000). Practical aspects of conducting a pragmatic randomised trial in primary care: patient recruitment and outcome assessment.. Br J Gen Pract.

[53] Sarris J (2007). Herbal medicines in the treatment of psychiatric disorders: a systematic review.. Phytother Res.

[54] Le CP (2001). Health and numbers: a problem-based introduction to biostatistics..

