# Supplementary material for: Naturopathic Care for Anxiety: A Randomized Controlled Trial ISRCTN78958974
Source: PLoS One. 2009 Aug 31;4(8):e6628. doi: 10.1371/journal.pone.0006628 (PMC2729375; doi:10.1371/journal.pone.0006628)
Supplement: Protocol S1 — Trial Protocol (0.45 MB DOC) [file pone.0006628.s002.doc]

**Treatment of anxiety amongst Canada Post workers: A randomized controlled trial**

**Clinical Trial Protocol**

**Version 1.0**

**September 22, 2005**

**Primary Investigator:**

**Ed Mills, DPH, MSc, PhD**

**Trial Coordinators:**

**Orest Szczurko, B.Sc., ND**

**Kieran Cooley, B.Sc., ND**

**Supervising Physician**

**Dan Perri, B.Sc.Phm., M.D., FRCPC**

**The Canadian College of Naturopathic Medicine**

**contact information:**

Orest M. Szczurko, The Canadian College of Naturopathic Medicine, 1255 Sheppard Ave. East, North York, Ontario, M2K 1E2, 416-498-1255, ext. 387, oszczurko@ccnm.edu

Table of Contents

1.0 Introduction 3

2.0 Scientific abstract 8

3.0 Proposed Study 10

3.1 Study Overview 10

3.1.1 Study Committee 10

3.1.2 Trial Site 11

3.1.3 Methodology 12

3.1.4 Design 12

3.1.5 Patient Selection 13

3.2 Inclusion/Exclusion Criteria 13

3.2.1 Inclusion Criteria 13

3.2.2 Exclusion Criteria 13

3.3 Timeline 14

4.0 Justification of Methodology 16

5.0 Feasibility 17

5.1 Time Line 18

5.2 Ethics 19

5.3 Funding and industry involvement 20

5.4 Relevance 21

Appendix A Beck Anxiety Inventory (BAI) 22

Appendix B Short Form - 36 (SF-36) 23

Appendix C Fatigue Questionaire 28

Appendix D Statement of Consent 30

Appendix E Administration of Funds 32

Appendix F Patient Questionaire and Recording Sheets 33

Appendix G References 45

# 1.0 Introduction

**Anxiety**

All humans experience fear and anxiety. Fear is an emotional, physiologic, and behavioral response to a recognized external threat (eg, an intruder, a runaway car). Anxiety is an unpleasant emotional state; but its causes are less clear. Anxiety is often accompanied by physiologic changes and behaviors similar to those caused by fear. [1]

Adaptive anxiety helps people prepare, practice, and rehearse so that their functioning is improved and helps them be appropriately cautious in potentially dangerous situations. Maladaptive anxiety causes distress and dysfunction. [1]

The causes of anxiety disorders are not fully known, but both physiologic and psychologic factors are involved. Physiologically, all thoughts and feelings may be understood as resulting from electrochemical processes in the brain, but this fact tells little about the complex interactions among the > 200 neurotransmitters and neuromodulators of the brain and about normal vs. abnormal arousal and anxiety. Psychologically, anxiety is viewed as a response to environmental stressors, such as the rupture of a significant relationship or exposure to a life-threatening disaster. [1]

Anxiety disorders are among the most prevalent psychiatric disorders in the Canada Post workers general population. Anxiety disorders are associated with considerable chronicity, morbidity, and disability [2,3]. Anxiety disorders impose high individual and social burden, tend to be chronic, and can be as disabling as somatic disorders. Compared with those who have other psychiatric disorders, people with anxiety disorders are high care utilizers who present to general practitioners more frequently than to psychiatric professionals, placing a strain upon the health care system and the Corporation drug benefit plan. The economic costs of anxiety disorders include psychiatric, and nonpsychiatric emergency care; hospitalization; prescription drugs; reduced productivity; absenteeism from work; and suicide [4].

Several studies have demonstrated the economic repercussions of widespread anxiety in the workplace. A data set from a large employer database has demonstrated both the medical and productivity costs of anxiety disorder. In the study, medical, pharmaceutical, absenteeism, short-term disability, and worker compensation records were collected during the year 2000 from 6 major employers. Employees diagnosed with anxiety disorders were significantly more likely to have additional diagnoses, use more services, require hospitalization, or visit the emergency room compared with the control group. In the final analysis, after controlling for differences in comorbidities, employees diagnosed with anxiety disorders had significantly higher medical costs, productivity costs, and total costs compared with the control group [5].

**Signs and Symptoms**

Anxiety can arise suddenly, as in panic, or gradually over many minutes, hours, or even days. Anxiety may last from a few seconds to years; longer duration is often associated with anxiety disorders. Anxiety ranges in intensity from barely noticeable qualms to complete panic, its most extreme form. One person's passion may be another's anxiety (eg, some find speaking before a group exhilarating, whereas others dread it), and the ability to tolerate anxiety varies from person to person [1,6].

Anxiety disorders can be so distressing and disruptive that depression may result. Alternatively, an anxiety disorder and depression may coexist, or depression may develop first, with symptoms and signs of an anxiety disorder developing later. [1,6]

Deciding when anxiety is so severe that it is a disorder depends on several variables. If anxiety is very distressing, interferes with functioning, and does not stop spontaneously within a few days, an anxiety disorder is present and merits treatment. [1,6]

Diagnosis of a specific anxiety disorder is based largely on its characteristic symptoms and signs. A family history of anxiety disorders (except posttraumatic stress disorder) is helpful, because many patients appear to have inherited a predisposition to the same anxiety disorders their relatives have as well as a general susceptibility to other anxiety disorders. [1,6]

Anxiety disorders must be distinguished from anxiety that occurs in many other psychiatric disorders, because they respond to different specific treatments. [1,6]

In Generalized Anxiety Disorder, the anxiety and worry are so great that they are difficult to control. The severity, frequency, or duration of the worry greatly exceeds what the situation, if it should occur, warrants. The focus of the worry is not restricted as it is in other psychiatric disorders (eg, to having a panic attack, being embarrassed in public, or being contaminated). Common worries include work responsibilities, money, health, safety, car repairs, and chores. A person with this disorder must also experience three or more of the following symptoms: restlessness, unusual fatigability, difficulty concentrating, irritability, muscle tension, and disturbed sleep. The course is usually fluctuating and chronic, with worsening during stress. [1,6]

**Conventional Treatment**

Benzodiazepines (Alprazolam, Chlordiazepoxide, Clonazepam, Clonazepate, Diazepam, Lorazepam, Oxazepam) in small to moderate doses are often prescribed, although sustained use can cause physical dependence. Consequently, the benzodiazepine should be tapered slowly rather than stopped abruptly, if it is to be discontinued. The relief obtained usually outweighs any mild adverse effects and the possibility of drug dependence. [1]

Buspirone is also effective for some patients, although its onset of effect takes about 2 wk, whereas benzodiazepines work within minutes. Buspirone does not cause dependency. Some antidepressants are also effective. [1]

The benefits of behavior therapy are limited because specifying anxiety triggers to which the person can be exposed is difficult. Relaxation and biofeedback may be of some help, although few studies have documented their efficacy. Insight-oriented psychotherapy has not been systematically studied in this disorder. [1]

**Naturopathic Treatments for Anxiety**

Herbs are powerful substances from which many pharmaceutical drugs have been formulated, yet there is a sense in the public domain that herbs are ‘natural’ and therefore harmless. In comparison, conventionally prescribed pharmaceutical drugs are often seen as fraught with risk and causing multiple adverse effects [7,8]. Evidence has shown, however, that herbal medicines can be harmful and that health care professionals need more education concerning the risks of these medicines [9-12]. It should also be noted that herbal medicines have a long tradition of use and many have been shown to be effective for a wide range of conditions. While numerous studies have been performed on Withania somnifera, there are only animal studies indicating its use as an anxiolytic. In an animal study assessing the anxiolytic and antidepressive actions of Withania somnifera compared to commonly prescribed pharmaceuticals, an extract of the root was administered orally to rats once daily for five days [13]. The results were compared to a group administered the benzodiazepine lorazepam for anxiolytic activity, and the tricyclic antidepressant imipramine for antidepressant investigation. Both the ashwagandha group and the lorzepaam group demonstrated reduced brain levels of a marker of clinical anxiety. Other similar studies confirm these results, lending support to the use of ashwagandha as an antistress adaptogen. [14-17]

A study showing the role of a Withania extract on CNS calcium antagonism, sheds some light on a potential mechanism of anxiolytic action.  With Withania extract supplementation the extracellular neuronal results obtained were consistent with calcium antagonistic properties, thereby counteracting excitation.  Calcium excitation plays a role in various psychiatric conditions including anxiety [18].

Diets high in stimulants have been linked to symptoms of anxiety. Reducing stimulants such as caffeine, chocolate, nicotine, refined sugars will reduce incidence and severity of anxiety [19]. Studies have shown that reduction in fat and alcohol also reduces the vulnerability to stress responses [20, 21].

Cognitive behavioural interventions such as diaphragmatic breathing have been shown to reduce anxiety levels and improve coping capability in stressful situations [22]. Therapies identifying negative stress coping patterns and replacing them with positive outlets are effective means of controlling and treating anxiety [23].

**Safety**

In addition to the criteria for measuring efficacy, there will also be assessment for harm and adverse effects that might arise from ingesting Whithania somnifera, or the Adult Multi One multivitamin. Participants will be monitored periodically by a naturopathic doctor and adverse events will be documented. If the participants feel as if their health is compromised in any way or the qualified investigator feels as if their health is compromised, they will immediately discontinue the trial and if necessary be referred to their medical doctor for further care. In addition, if any significant health issues arise independent to the trial, the participants will also discontinue the trial and be referred to their medical doctor. If it is found that the health of any of the participants is compromised in any way, they will be immediately pulled from the study. Approval has been sought and granted from the ethics review board of the Canadian College of Naturopathic Medicine. Approval is also being requested from Health Canada and is necessary before beginning the study. It should be noted that Withania somifera, and the Adult Multi One multivitamin are already on the market and any determination of harm is crucial for public safety and should be disseminated rapidly and effectively. If on the other hand, any efficacy is found then the public will also be so informed giving an alternative option for treatment of anxiety perhaps without increased risk of complications or sedation.

# 2.0 Scientific abstract

**Background:** Anxiety disorders are among the most prevalent psychiatric disorders in the Canada Post workers general population, yet there is a lack of information with regards to safety and efficacy of combined naturopathic approache for anxiety. There is a body of literature that supports usage of some herbs, dietary advice, exercise, and cognitive techniques in the treatment of anxiety, however there is very little research on the combined approach in a workplace setting. We aim to evaluate the efficacy and safety of dietary advice, exercise, cognitive counselling, Withania sominfera and a Adult Multi One multivitamin on common indicators of anxiety

**Design:** Phase II Randomised parallel clinical trial.

**Objective:** To determine if dietary modification, exercise, counselling, Withania somnifera, and a multi vitamin decreases the anxiety scores in a working population at Canada Post.

**Null hypothesis:** Dietary modification, exercise, counselling, Withania somnifera, and a multi vitamin has no effect on anxiety scores.

**Participants:** 80 Canada Post employees who are who are experiencing the symptoms anxiety (who score over 10 points on the Beck Anxiety Inventory) will be randomised to two groups: one receiving dietary modification, exercise, counselling, Withania somnifera, and a multi vitamin (active group), and one receiving dietary modification, exercise, counselling, and placebo (control group).

**Setting:** Clinical trial will be conducted at the Canada Post Gateway Processing Plant.

**Intervention:** Baseline assessment of both groups will include blood pressure, orthostatic blood pressure, and completion of the Beck Anxiety Inventory (BAI), SF-36, and The Fatigue Questionnaire to determine severity of anxiety symptoms. The active group will receive instruction on stress reduction techniques including diaphragmatic breath control, encouragement to exercise, counselling to identify and eliminate negative stress coping patterns, dietary counselling to ensure proper nutritional content of foods, and reduce reactive hypoglycaemia, Withania somnifera, and a multi vitamin for 12 weeks. The control group will receive instruction on stress reduction techniques including diaphragmatic breath control, encouragement to exercise, counselling to identify and eliminate negative stress coping patterns, and placebo. Every 4 weeks (day 28, day 56) both groups will again have their sitting and orthostatic blood pressure checked, and complete the BAI, SF-36, and The Fatigue Questionnaire. On day 84, a final assessment of sitting and orthostatic blood pressure, BAI, SF-36, and The Fatigue Questionnaire completion will take place.

**Main Outcome Measures:** ***The Beck Anxiety Inventory (BAI)*** is designed to discriminate anxiety from depression in individuals. The BAI consists of 21 items, each describing a common symptom of anxiety. The items are summed to obtain a total score that can range from 0-63. The scale has high internal consistency. ***The short form –36*** (SF-36) questionnaire is a self-administered, 36-item questionnaire that measures health-related quality of life in eight domains: 1) physical functioning, by measuring the ability to perform a variety of daily activities and tasks that require physical effort (10 items); 2) role limitations due to physical problems(4 items); 3)role limitations due to emotional problems (3 items); 4) vitality, measuring perceived level of energy and fatigue (4 items); 5) freedom from bodily pain (2 items); 6)social functioning (2 items); 7) mental health, by measuring both negative and positive emotional states (5 items) and 8)general health perceptions (6 items). Both physical and mental summary scores can be obtained. Each domain is scored separately from 0 (lowest level) to 100 (highest level). Two summary scores can be calculated from information obtained in the 8 domains- Physical Function and Mental Health Summary Scores. ***The Fatigue Questionnaire*** is an 11-item instrument developed for use in population studies to measure physical and mental aspects of fatigue, a feature commonly observed in many somatizing patients. Each item has 4 response options, with high scores corresponding to high levels of fatigue.

**Relevance:** Many patients seek alternatives to conventional anxiety treatments. To date, no study has evaluated the potential for the combined efficacy of dietary, counselling, exercise, Withania somnifera and a Adult Multi One multivitamin to effectively treat anxiety. Knowledge of the efficacy, safety or risk of natural health products, especially one already on the market is important for physicians and the public in order to make informed decisions.

# 3.0 Proposed Study

This randomised, blinded, controlled trial will evaluate the efficacy and safety of dietary and behavioural counselling, exercise, Withania somnifera and a Adult Multi One multivitamin to effectively treat anxiety. This will be an intervention trial using treatments and products widely available on the market, and an indistinguishable placebo. The study sample will be randomised into two arms: treatment and control. The study population will consist of 80 Canada Post Employees.

## 3.1 Study Overview

### 3.1.1 Study Committee

**Primary Investigator:**

Ed Mills, Canadian College of Naturopathic Medicine, 1255 Sheppard Ave. East, North York, Ontario, M2K 1E2. 416-4981255 ext. 324, [emills@ccnm.edu](mailto:wmills@ccnm.edu)

**Study Co-ordinators:**

Orest Szczurko, Canadian College of Naturopathic Medicine, 1255 Sheppard Ave. East, North York, Ontario, M2K 1E2. 416-4981255 ext. 317, [oszczurko@ccnm.edu](mailto:oszczurko@ccnm.edu)

Kieran Cooley, Canadian College of Naturopathic Medicine, 1255 Sheppard Ave. East, North York, Ontario, M2K 1E2. 416-4981255 ext. 317, [kcooley@ccnm.edu](mailto:kcooley@ccnm.edu)

**Supervising Physician:**

Dr. Dan Perri B.Sc.Phm., M.D., FRCPC. Unit 14 – 289 Plains Road West, Burlington, Ontario, L7T 1G1 (905) 572-3433 dan.perri@utoronto.ca

### 3.1.2 Trial Site

The trial shall take place at the Canada Post Gateway Processing Plant, 4567 Dixie Road, Mississauga Ontario, L4W 1S2 under the supervision of the four investigators listed above.

### Methodology

Setting and Participants: The setting for this study will be the Gateway Processing Plant of the Canada Post Corporation in Mississauga. The study will take place from September to December, and will involve Canada Post employees and members of the Canadian Union of Postal Workers (CUPW). Workers will primarily be from the Gateway plant, however a minority of the study population will be from other Canada Post facilities. There will be a mix of workers, from day to evening to night shift letter and package sorters, to outdoor letter carriers and drivers. The study population will be male and female, in the age groups from 18 to 65.

Sampling and Recruitment: Recruitment for the study will be facilitated by Canada Post Corporation and CUPW representatives. Posters and information sheets will be posted and distributed throughout eligible sites. Interested parties will be encouraged to contact the Naturopathic Doctors directly, either by phone or e-mail and provide their name, phone number, preferred time of contact, and mailing address. Interested participants will be given an Information Kit, which will include: a sample consent form, a Backgrounder explaining the purpose of the study, a description of Naturopathic Modalities, a question and answer sheet regarding the study, contact list and phone numbers of the Naturopathic Doctors, Canada Post, and CUPW representatives involved in the study, and the Naturopathic Doctors business cards. The two trial coordinators will also conduct brief information sessions at the pre-shift meetings. Interested participants will be encouraged to ask questions to their Canada Post or CUPW representatives, and directly to the Naturopathic Doctors. Following the mailing of the Information Kit, interested participants will be contacted by telephone, screened for stress and or anxiety and availability for the entire duration of the study, and scheduled for a 1 hour initial consultation in a designated room at the Canada Post Gateway Processing Plant. At this consultation, any outstanding questions will be answered by the Naturopathic Doctors, a waiver and consent form will be signed and witnessed, and the intake interview and physical exam to evaluate the eligibility for the study will take place. At the end of the initial visit, potential participants will be instructed to complete the Beck Anxiety Inventory, S-90-R, SF-36, and a Fatigue Questionnaire. A minimal score on the Beck Anxiety Inventory, as well as absence of any contraindications found on the initial intake, will be used to determine eligibility for the study.

At the conclusion of the initial visit, participants will receive the Diet, Absenteeism, Medication and Therapies Diary (Appendix I) chart to complete at home over the next week. Eligible participants will be randomly assigned to either the control or treatment groups, and will be booked for further appointment times.

On subsequent visits, both the control and intervention group patients will receive treatments including cognitive behavioural stress reduction, behavioral psychotherapy, examination of sleep patterns and treatment for any insomnia. Both groups will be encouraged to engage in an active exercise routine. The active group will also receive daily good quality multi vitamin (Ultra Balance III by Douglas Labs) and *Withania somnifera* (300 mg standardized to 1.5% Withanolides, bid) (Ashwagandha by Douglas Labs). The control group will receive placebo identical in look and frequency of dosage to the treatment group. On subsequent visits, the active group will receive lifestyle and diet counseling, and will continue with behavioral psychotherapy. Active group patients will also receive acupressure massage for at least 15 minutes once per week.

Both the active and control arm will attend weekly 30 minute treatments, for a total of 12 treatments over 12 weeks. Both groups will complete the Beck Anxiety Inventory, S-90-R, SF-36, Fatigue Questionnaire, and the Perceived Benefit of Treatment questionnaires at 4, 8, and 12 weeks. At these times, the sitting and orthostatic blood pressures will also be recorded. Both groups will also hand in the Pain Diet, Absenteeism, Pain Medication and Therapies Diary every week.

Study period is 12 weeks. Clinical evaluations will be performed at:

- Baseline (Visit 1 - Day 1)
- Interim for the treatment groups: (Visit 2 - Day 7, Visit 3- Day 14, Visit 4 – Day 21, Visit 4 – Day 28, Visit 5 – Day 35, Visit 6 – Day 42, Visit 7 – Day 49, Visit 8 – Day 56, Visit 9 – Day 63, Visit 10 – Day 70, Visit 11 – Day 77)
- Interim for the control groups: (Visit 2 - Day 7, Visit 3- Day 14, Visit 4 – Day 21, Visit 4 – Day 28, Visit 5 – Day 35, Visit 6 – Day 42, Visit 7 – Day 49, Visit 8 – Day 56, Visit 9 – Day 63, Visit 10 – Day 70, Visit 11 – Day 77)
- End of Treatment (Visit 12 - Day 84), Questionnaires and Medication diaries filled in for the last time

Follow-up data will be collected at 2, 6, and 12 months post treatment via a mailed questionnaire package containing the Beck Anxiety Inventory, S-90-R, SF-36, Fatigue Questionnaire, and the Perceived Benefit of Treatment questionnaires, as well as the Diet, Absenteeism, Medication and Therapies Diary. Non-responders will receive reminders by post. If this is unsuccessful, the patients will be telephoned.

### 3.1.4 Design

Single blinded, randomised, controlled intervention trial..

### 3.1.5 Patient Selection

80 healthy subjects irrespective of ethnicity, aged 18 to 65 that are self-selected through response to local ads and information talks through Canada Post Centres in Toronto. Contraindications will be ruled out first and informed consent must be received from all participants before eligibility is assigned. Mild anxiety must be exhibited, and a score of 10 must be achieved on the Beck Anxiety Inventory administered on intake in order for patients to qualify.

## 3.2 Inclusion/Exclusion Criteria

### 3.2.1 Inclusion Criteria

1. Males and females aged 18-65 that are Canada Post employees and members of Canadian Union of Postal Workers (CUPW).

2. Mentally competent subjects able to adhere to the given protocol and treatments administered as interventions.

3. Self-selected candidates identifying themselves to suffer from the symptoms of stress and anxiety for a period of 6 weeks or more and a score of at least 10 on the BAI at the pre-study intake.

4. Normal on physical examination at the pre-study intake, and in the case of abnormalities the medical practitioner considers them to be clinically insignificant.

5. Written and informed consent.

6. The potential candidate must have a family doctor that they have seen in the last 12 months.

### 3.2.2 Exclusion Criteria

1. Mentally or physically incapacitated such that informed consent cannot be obtained.

2. Any history or other condition which the study physician regards as clinically significant to the study (including allergies or sensitivities to withanolides or multivitamins).

3. A major illness considered to be clinically significant by the study physician within 3 months of the study start date.

4. Current participation in another intervention trial.

5. Pregnancy or intent to become pregnant in the next 6 months.

6. Medication at doses that is contraindicated with herb/multi-vitamin. (specifically daily use of benzodiazepene class drugs.)

7. Current alcoholism or substance abuse (sedative)

8. Current history of tumors.

9. Any current serious disorders determined to be clinically significant to the study.

.

## 3.3 Timeline

1. Labelled as day 0, all potential candidates will be screened for inclusion/exclusion criteria by being asked to read and sign the informed consent, fill in questionnaires including the BAI, be clinically assessed, have their blood pressure taken, and have urine tests to assess for proper liver and kidney function.

1. On day 1, participants that have fulfilled all eligibility criteria and completed the consent form will be randomised into either treatment or control groups using a random number generator by the research assistant who will label the containers of placebo, *Withania somnifera* and multivitamin respectively.
2. Both treatment and placebo groups shall receive the visually identical capsules by the study co-ordinator with instructions on the dosage schedule.
3. Both groups shall take their medications twice daily, one capsule in the morning and one in the evening prior to meals.
4. The treatment shall last 90 days.
5. On day 1, subjects will be clinically assessed, have their blood pressure taken, be interviewed, and complete the SCL-90R, SF-36 and fatique questionnaires. Medication usage will be monitored and compliance to the interventions will be assessed.
6. On day 7, subjects will receive cognitive–behavioral therapy, dietary advice and stress reduction techniques. They will continue these treatment sessions 1 time per week until the completion of the study. (Day 14, 21, 28, 35, 42, 49, 56, 63, 70, 77 and 84)
7. On day 8, subjects in the active group will receive acupressure massage in the treatment of anxiety. They will continue these treatment sessions 1 time per week until the completion of the study. . (Day 15, 22, 30, 38, 45, 52, 60, 68, 75, 82 and 90)
8. On day 28, subjects will be clinically assessed, have their blood pressure taken, be interviewed, and complete the SCL-90R, SF-36 and fatigue questionnaires
9. On day 56, subjects will be clinically assessed, have their blood pressure taken, be interviewed, and complete the SCL-90R, SF-36 and fatigue questionnaires
10. On day 84, subjects will be clinically assessed, have their blood pressure taken, be interviewed, and complete the SCL-90R, SF-36 and fatigue questionnaires

Adherence to the schedule and adverse effects will be assessed at patient interviews at each stage of the study. In addition, subjects will have access by phone or visit to the supervising physician if they feel their health is being compromised in any way during the study. As a way of measuring and promoting compliance, subjects will be given study calendars and asked to return their unfinished pills.

.

**Outcome measurements**

We will determine if naturopathic medical interventions (*Withania somnifera* extract, daily multi-vitamin, dietary modifications, cognitive-behavioral exercises, relaxation techniques and acupressure massage) reduces the incidence of specific symptoms as measured by the Beck Anxiety Inventory as shown in appendix A. This scale has been independently validated.

**Sample size determination**

Assuming a normal distribution with variances between both populations (pre and post treatment) being essentially equal, we made our power size calculations using Jan deLeeuw's Web-Based Statistics: The Study of Stability in Variation[24]. To obtain a power of 80% with a two sided 5% significance level we would need 54 participants to complete this two-treatment parallel-design study. This will enable the detection of a 40% difference in the Beck Anxiety Inventory rating scale, an amount considered to be clinically significant. Assuming a drop out rate of 30% we will need 78 subjects to commence the trial so that 54 will complete the trial.

**Statistical Analysis**

# Data recovered from the menopause rating scale of each participant will be analysed with the chi-square test and the student’s t-test. Statistical analysis will be conducted on code numbered subjects where treatment protocol is unknown. In other words statistical analysis will be blinded as to the results.

# 4.0 Justification of Methodology

The study trial shall involve 78 subjects as determined above. The trial shall last 84 days (12 weeks) which is greater than the mean treatment time for most trials reviewed and we feel enough time to determine whether a clinically significant effect will be produced. Longer duration would not likely show much more increased effect but rather might jeopardise the results through loss of participation and or incorporation of other treatments. The extended duration will ensure all active substances are metabolised and will allow for more accurate data collection on the effects of naturopathic medical treatments.

We employed a controlled design, rather than the conventional before/after design employed by many intervention studies as we wish to assess not only activity in the treatment group but also to determine if any change is significantly different from a control group not receiving the intervention. The absence of a control group would make it impossible to determine if variability in metabolism within individuals has overshadowed the potential impact of naturopathic medical treatments on anxiety symptoms. Examples of factors that could influence variability within individuals include changes in stimulant use, sleeping habits, compliance to treatment interventions, exercise levels and environmental stressors that may occur over the course of the study. Traditional drug intervention studies that are not controlled are inherently susceptible to bias and it is very difficult to determine if the results have been skewed.

# 5.0 Feasibility

**Study committee:**

Ed Mills DPH, MSc, PHd (cand) is the Director of Research in the Canadian College of Naturopathic Medicine. He has extensive experience in research methodology and design and will be overseeing the trial throughout its duration.

Orest Szczurko BSc., ND is a licensed naturopathic doctor and is a Clinical Trial Coordinator at the Canadian College of Naturopathic Medicine. He has experience in trial design and conduct, drug efficacy studies, interviewing skills, and clinical skills.

Kieran Cooley, BSc. ND is a licensed naturopathic doctor and is a Clinical Trial Coordinator at the Canadian College of Naturopathic Medicine. He has experience in trial design and conduct, interviewing skills and clinical skills.

Dan Perri B.Sc.Phm., M.D., FRCPC is a medical doctor with extensive clinical and research experience.

**Setting:**

The Gateway Processing Plant is an ideal institution from which to adequately supervise and conduct the study. Study participants will be volunteers recruited from Canada Post employees through advertisements in corporate newsletters, information and talks led by the trial investigators. Participants will be fully informed of the treatment they may receive and must give informed consent before enrolment.

## 5.1 Time Line

The subjects will be directly involved in the study for twelve weeks. We intend on recruiting for the trial immediately upon approval of the trial by Health Canada. Recruitment should take three to six weeks and if possible be complete before the end of October. We will commence with treatment after recruitment of 80 eligible participants, which is projected to early November. The trial itself will be completed by the end of January 2006. Data analysis will ensue followed by publication in a peer reviewed article sometime in the Winter of 2006.

## 5.2 Ethics

We have passed this study through the internal review board at CCNM for ethics approval, please see the Research Ethics Board Attestation. Informed consent will be obtained from all enrolled subjects, please see Appendix D for Informed Consent forms. Management and assessment of health concerns will be under the direction of Dr. Dan Perri MD.

## 5.3 Funding and industry involvement

Support for the study has been generously provided by Canada Post Corporation, Canadian Union of Postal Workers, and Swiss Herbals through the Research Department of the Canadian College of Naturopathic Medicine. Funding to the college is under the control of the chief financial officer at The Canadian College of Naturopathic Medicine, see appendix E. Beyond the confines of this trial, none of the investigators, nor the supervising physician involved have any ties or relationships with Swiss Herbals.

This trial was registered with the International Standard Randomized Clinical Trial Registry (trial registration number: ISRCTM78958974 available at <http://www.controlled-trials.com/isrctn/search.html?srch=ISRCTN78958974&sort=3&dir=desc&max=10>). Registration of this trial took place after the trial had been completed due to a number of complications with previous submissions and delays in the registration process. We additionally recognize a lack of knowledge on the part of the authors with regards to clinical trial registration – initially it was our understanding that because the trial was taking place within a workplace setting, registration was not necessary. After recognizing our error and misunderstanding, we proceeded with a registration attempt. We declare that this delay was **in no part** due to conflicts of interest, or an attempt to avoid open knowledge of the existence, conduct or results of the trial.

- 1. **Relevance**

Anxiety disorders are among the most prevalent psychiatric disorders in the Canada Post workers general population. Anxiety disorders are associated with considerable chronicity, morbidity, and disability. The economic costs of anxiety disorders include psychiatric, and nonpsychiatric emergency care; hospitalization; prescription drugs; reduced productivity; absenteeism from work; and suicide. We aim to evaluate the efficacy of typical Naturopathic treatment on anxiety. This will also lay the groundwork for an assessment of the cost effectiveness of Naturopathic Medicine in the treatment of anxiety in the work place.

.

# APPENDIX A BECK ANXIETY INVENTORY

Below is a list of common symptoms of anxiety. Please carefully read each item in the list. Indicate how much you have been bothered by that symptom during the past month, including today, by circling the number in the corresponding space in the column next to each symptom.

|  | Not At All | Mildly but it didn’t bother me much. | Moderately - it wasn’t pleasant at times | Severely – it bothered me a lot |
| --- | --- | --- | --- | --- |
| Numbness or tingling | 0 | 1 | 2 | 3 |
| Feeling hot | 0 | 1 | 2 | 3 |
| Wobbliness in legs | 0 | 1 | 2 | 3 |
| Unable to relax | 0 | 1 | 2 | 3 |
| Fear of worst happening | 0 | 1 | 2 | 3 |
| Dizzy or lightheaded | 0 | 1 | 2 | 3 |
| Heart pounding/racing | 0 | 1 | 2 | 3 |
| Unsteady | 0 | 1 | 2 | 3 |
| Terrified or afraid | 0 | 1 | 2 | 3 |
| Nervous | 0 | 1 | 2 | 3 |
| Feeling of choking | 0 | 1 | 2 | 3 |
| Hands trembling | 0 | 1 | 2 | 3 |
| Shaky / unsteady | 0 | 1 | 2 | 3 |
| Fear of losing control | 0 | 1 | 2 | 3 |
| Difficulty in breathing | 0 | 1 | 2 | 3 |
| Fear of dying | 0 | 1 | 2 | 3 |
| Scared | 0 | 1 | 2 | 3 |
| Indigestion | 0 | 1 | 2 | 3 |
| Faint / lightheaded | 0 | 1 | 2 | 3 |
| Face flushed | 0 | 1 | 2 | 3 |
| Hot/cold sweats | 0 | 1 | 2 | 3 |
| Column Sum |  |  |  |  |

## Scoring - *Sum each column. Then sum the column totals to achieve a grand score. Write that score here _______*

## Appendix B SF-36

Your Health and Well-Being

**This survey asks for your views about your health. This information will help keep track of how you feel and how well you are able to do your usual activities. *Thank you for completing this survey!***

**For each of the following questions, please mark an**  **in the one box that best describes your answer.**

1. In general, would you say your health is:

| Excellent | Very good | Good | Fair | Poor |
| --- | --- | --- | --- | --- |
|  |  |  |  |  |
| 1 | 2 | 3 | 4 | 5 |

**2. Compared to one year ago, how would you rate your health in general now**?

| Much better  now than one  year ago | Somewhat  better  now than one  year ago | About the  same as  one year ago | Somewhat  worse  now than one  year ago | Much worse  now than one  year ago |
| --- | --- | --- | --- | --- |
|  |  |  |  |  |
| 1 | 2 | 3 | 4 | 5 |

|  | Yes,  limited  a lot | Yes,  limited  a little | No, not  limited  at all |
| --- | --- | --- | --- |
|  |  |  |  |
| a Vigorous activities, such as running, lifting  heavy objects, participating in strenuous sports  1  2  3 | | | |
| b Moderate activities, such as moving a table, pushing  a vacuum cleaner, bowling, or playing golf  1  2  3 | | | |
| c Lifting or carrying groceries  1  2  3 | | | |
| d Climbing several flights of stairs  1  2  3 | | | |
| e Climbing one flight of stairs  1  2  3 | | | |
| f Bending, kneeling, or stooping  1  2  3 | | | |
| g Walking more than a kilometre  1  2  3 | | | |
| h Walking several hundred metres  1  2  3 | | | |
| i Walking one hundred metres  1  2  3 | | | |
| j Bathing or dressing yourself  1  2  3 | | | |

3. The following questions are about activities you might do during a typical day. Does your health now limit you in these activities? If so, how much?

4. During the past 4 weeks, how much of the time have you had any of the following problems with your work or other regular daily activities as a result of your physical health?

|  | All of  the time | Most of  the time | Some of  the time | A little of  the time | None of  the time |
| --- | --- | --- | --- | --- | --- |
|  |  |  |  |  |  |
| a Cut down on the amount of  time you spent on work or  other activities  1  2  3  4  5 | | | | | |
| b Accomplished less than you  would like  1  2  3  4  5 | | | | | |
| c Were limited in the kind of  work or other activities  1  2  3  4  5 | | | | | |
| d Had difficulty performing the  the work or other activities (for  example, it took extra effort)  1  2  3  4  5 | | | | | |

5. During the past 4 weeks, how much of the time have you had any of the following problems with your work or other regular daily activities as a result of any emotional problems (such as feeling depressed or anxious)?

|  | All of  the time | Most of  the time | Some of  the time | A little of  the time | None of  the time |
| --- | --- | --- | --- | --- | --- |
|  |  |  |  |  |  |
| a Cut down on the amount of  time you spent on work or  other activities  1  2  3  4  5 | | | | | |
| b Accomplished less than you  would like  1  2  3  4  5 | | | | | |
| c Did work or other activities  less carefully than usual  1  2  3  4  5 | | | | | |

6. During the past 4 weeks, to what extent has your physical health or emotional problems interfered with your normal social activities with family, friends, neighbors, or groups?

| Not at all | Slightly | Moderately | Quite a bit | Extremely |
| --- | --- | --- | --- | --- |
|  |  |  |  |  |
| 1 | 2 | 3 | 4 | 5 |

7. How much bodily pain have you had during the past 4 weeks?

| None | Very mild | Mild | Moderate | Severe | Very severe |
| --- | --- | --- | --- | --- | --- |
|  |  |  |  |  |  |
| 1 | 2 | 3 | 4 | 5 | 6 |

8. During the past 4 weeks, how much did pain interfere with your normal work (including both work outside the home and housework)?

| Not at all | A little bit | Moderately | Quite a bit | Extremely |
| --- | --- | --- | --- | --- |
|  |  |  |  |  |
| 1 | 2 | 3 | 4 | 5 |

9. These questions are about how you feel and how things have been with you during the past 4 weeks. For each question, please give the one answer that comes closest to the way you have been feeling. How much of the time during the past 4 weeks…

|  | All of  the time | Most of  the time | Some of  the time | A little of  the time | None of  the time |
| --- | --- | --- | --- | --- | --- |
|  |  |  |  |  |  |
| a Did you feel full of life?  1  2  3  4  5 | | | | | |
| b Have you been very nervous?  1  2  3  4  5 | | | | | |
| c Have you felt so down in the  dumps that nothing could  cheer you up?  1  2  3  4  5 | | | | | |
| d Have you felt calm and  peaceful?  1  2  3  4  5 | | | | | |
| e Did you have a lot of energy?  1  2  3  4  5 | | | | | |
| f Have you felt downhearted  and depressed?  1  2  3  4  5 | | | | | |
| g Did you feel worn out?  1  2  3  4  5 | | | | | |
| h Have you been happy?  1  2  3  4  5 | | | | | |
| i Did you feel tired?  1  2  3  4  5 | | | | | |

10. During the past 4 weeks, how much of the time has your physical health or emotional problems interfered with your social activities (like visiting with friends, relatives, etc.)?

| All of  the time | Most of  the time | Some of  the time | A little of  the time | None of  the time |
| --- | --- | --- | --- | --- |
|  |  |  |  |  |
| 1 | 2 | 3 | 4 | 5 |

11. How TRUE or FALSE is each of the following statements for you?

|  | Definitely  true | Mostly  true | Don’t  know | Mostly  false | Definitely  false |
| --- | --- | --- | --- | --- | --- |
|  |  |  |  |  |  |
| a I seem to get sick a little easier than other people  1  2  3  4  5 | | | | | |
| b I am as healthy as  anybody I know  1  2  3  4  5 | | | | | |
| c I expect my health to  get worse  1  2  3  4  5 | | | | | |
| d My health is excellent  1  2  3  4  5 | | | | | |

*Thank you for completing these questions!*

## Appendix c Fatigue questionnaire

Below you will find 20 statements regarding your health. With these statements we hope to get an impression of your health within the **past 2 weeks.**

If you find that the statement very **true**, put an **X** in the appropriate box, like this:

I feel relaxed yes, that is true ******** no, that is not true

If you find the statement **not true**, put an **X** in the appropriate box, like this:

I feel relaxed yes, that is true ******** no, that is not true

If you find the statement to be true sometime, and not true at others, place an X in the box in accordance to how you have felt

I feel relaxed yes, that is true ******** no, that is not true

1. I feel tired yes, that is true ******** no, that is not true

2. I feel very active yes, that is true ******** no, that is not true

3. Thinking requires effort yes, that is true ******** no, that is not true

4. Physically, I feel exhausted yes, that is true ******** no, that is not true

5. I feel like doing all kinds of nice things yes, that is true ******** no, that is not true

6. I feel fit yes, that is true ******** no, that is not true

7. I do quite a lot within a day yes, that is true ******** no, that is not true

8. When I am doing something

I can concentrate quite well yes, that is true ******** no, that is not true

9. I feel weak yes, that is true ******** no, that is not true

10.I don’t do much during the day yes, that is true ******** no, that is not true

11.I can concentrate well yes, that is true ******** no, that is not true

12. I feel rested yes, that is true ******** no, that is not true

13. I have trouble concentrating yes, that is true ******** no, that is not true

14. Physically I feel I am

in a bad condition yes, that is true ******** no, that is not true

15. I am full of plans yes, that is true ******** no, that is not true

16. I get tired quickly yes, that is true ******** no, that is not true

17. I have a low output yes, that is true ******** no, that is not true

18. I feel no desire to do anything yes, that is true ******** no, that is not true

19. My thoughts easily wander yes, that is true ******** no, that is not true

20. Physically I feel I am in good shape yes, that is true ******** no, that is not true

## Appendix D Statement of Consent


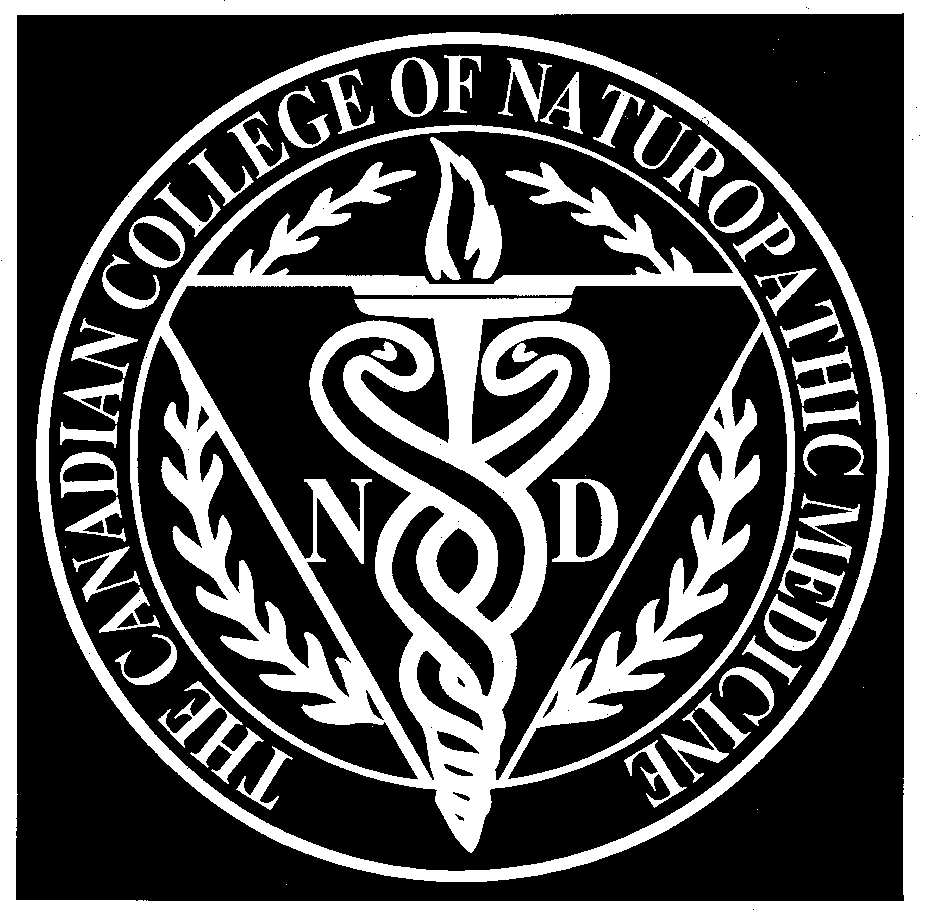


The Canadian College of Naturopathic Medicine Department of Research, 1255 Sheppard Ave. E., Toronto, Ontario, M5K-1E5

**Statement of Informed Consent**

I agree to participate in a study investigating Naturopathic Treatments for Stress and Anxiety. I understand that the purposes of this study are for research and all the varieties of treatments involved may not adequately address my stress and anxiety. This study is not intended to replace or act as a substitute for any other treatment in which I am currently engaged.

The following points have been explained to me: The purpose of this research is to determine the effectiveness of naturopathic treatment for stress and anxiety. The benefits I may expect from the study are: (a) naturopathic treatment for stress and anxiety. (b) an opportunity to contribute to scientific research, (c) to help those who come after me and (d) an appreciation of research on health.

The procedure will be as follows; on day one I shall attend the clinic where I will be given a physical exam, asked questions about my medical history and I will be provided 3 questionnaires to answer regarding stress and anxiety, fatigue, and quality of life assessment.

The researchers have pointed out to me that following the physical exam, intake, and medical history there is a possibility that I may not be chosen to participate in the study based on their findings. The reasons for this are purely health related and are in place for the benefit of the participants as well as to ensure that all participants adequately experience stress and anxiety.

If am chosen to participate in the study, I will be randomly assigned to a treatment or control group in a manner similar to the flipping of a coin. Follow-up treatments will be scheduled at this time, where I will receive supplements, lifestyle suggestions, and varying naturopathic treatments that may include nutritional counselling, stress reduction techniques, and acupressure massage. Follow up visits will be weekly. I will also receive a diet diary and log by which I will monitor usage of any medications. I shall attend the clinic again every week for follow up visits, every 4 weeks I will be provided 3 questionnaires to answer concerning my symptoms and progression of treatment. I am responsible for following the study protocol for a period of 12 weeks in total.

In the case where I feel my health is in any way compromised or my stress and anxiety are not being addressed during the study, I shall contact either Orest Szczurko, or Kieran Cooley and report symptoms and concerns that I feel are warranted.

INITIAL ______

My responses will not be associated with my name; instead, my name will be converted to an anonymous code number when the researchers store the data. All records created, and data collected are strictly confidential and will be kept at the Canadian Colledge of Naturopathic Medicine. Only the clinicians associated with the study will see my responses.

This consent form will be detached from the questionnaire and stored separately. If I have any other questions or concerns, I can address them to the study coordinator Orest Szczurko, or the secondary clinician Kieran Cooley using the contact information below:

Orest Szczurko, Canada Post Trial Coordinator, Canadian College of Naturopathic Medicine, 416-722-9136, [oszczurko@ccnm.edu](mailto:oszczurko@ccnm.edu)

Kieran Cooley, Canada Post Trial Investigator, Canadian College of Naturopathic Medicine, 416-407-1331, [kcooley@ccnm.edu](mailto:kcooley@ccnm.edu)

This study is funded by the Canada Post Corporation (CPC) and the Canadian Union of Postal Workers (CUPW) and their involvement is purely monetary.

This study has been approved by the Canadian College of Naturopathic Medicine Research Ethics Board, Health Canada and the Natural Health Products Directorate Ethics Board.

I volunteer to follow the supplement protocol. Following the study, I give the researchers permission to review my absenteeism records. This will be done in strict confidentiality, and will serve to evaluate the effectiveness of the treatment.

I have read the above statements, understand them, and would like to participate in this study.

I understand that my participation is entirely voluntary: and I may leave the experiment at any time.

**I have read the above statements, understand them, and would like to participate in this study.**

Participant’s Printed Name _______________________________________________________

Participant’s Signature __________________________________________________________

Date: ________________________________________________________________________

**FOR OFFICE USE ONLY:**

Experimenter's Printed Name ____________________________________________

Experimenter's Signature _______________________________________________

Participant Number ____________________________________________________

INITIAL _____

## Appendix E Administration of Funds

**Administration of Funds**

Name: Ms. Anne Kim

Title: Chief Financial Officer

Telephone: 416-498-1255 ext 221

Institution: The Canadian College of Naturopathic Medicine

Address: 1255 Sheppard Ave. East, North York, Ontario, M2K 1E2

Fax: 416-498-1611

Email: [akim@ccnm.edu](mailto:akim@ccnm.edu)

## Appendix F Patient questionaire and Recording sheets

#
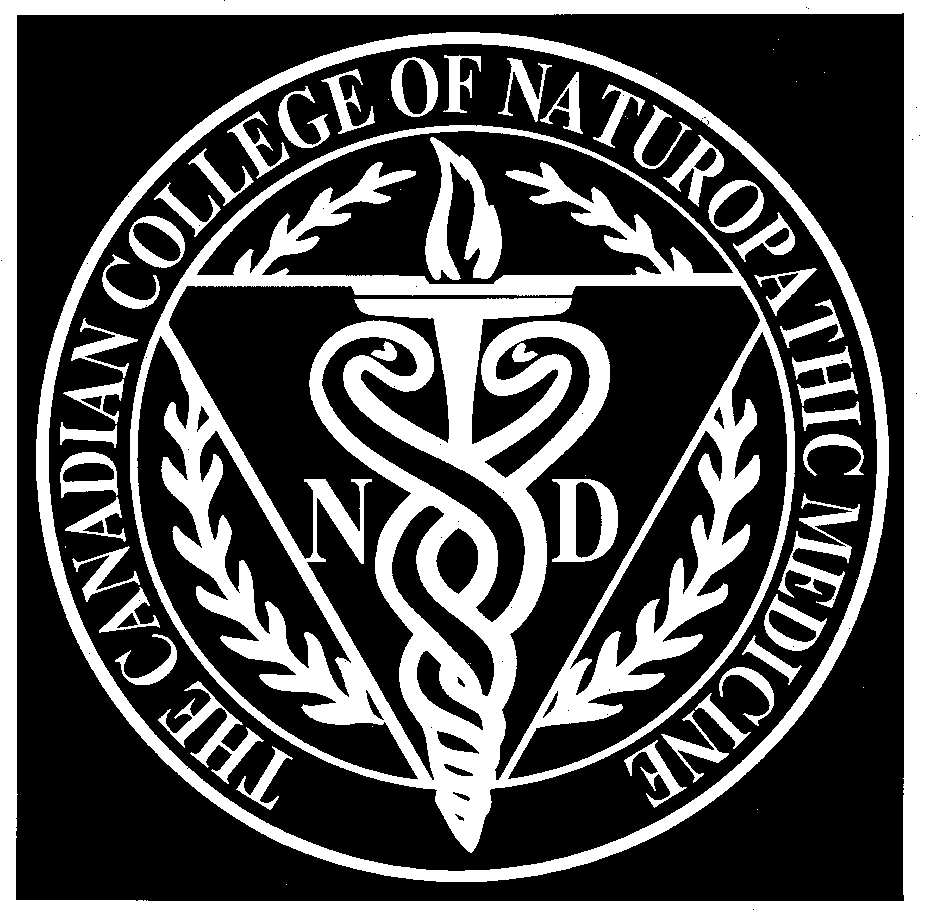
THE CANADIAN COLLEGE OF NATUROPATHIC MEDICINE

**Treatment of anxiety amongst Canada Post workers: A randomized controlled trial**

# Principal investigator: Ed Mills

# Study Co-ordinators: Orest Szczurko and Kieran Cooley

# Date: ____________________________

# Subject: ____________________________

# Phone Number: ____________________________

# Email: ____________________________

# Study Number: ____________________________

#
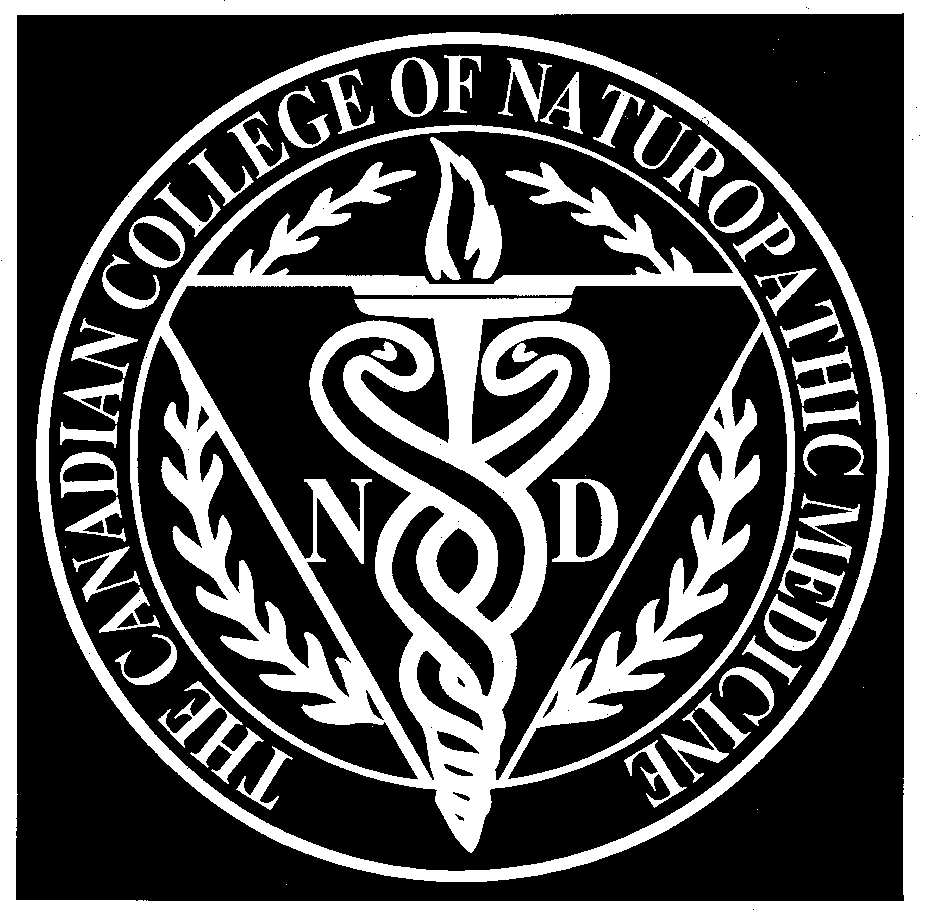
THE CANADIAN COLLEGE OF NATUROPATHIC MEDICINE

**Treatment of anxiety amongst Canada Post workers: A randomized controlled trial**

# Principal investigator: Ed Mills

# Study Co-ordinators: Orest Szczurko and Kieran Cooley

# Schedule of events (day)

| Activity | 0 | 1 | 28 | 56 | 84 |
| --- | --- | --- | --- | --- | --- |
| Inclusion/Exclusion Criteria | X |  |  |  |  |
| BAI | X |  | X | X | X |
| Urine Test to rule out Gross liver and Kidney Pathologies | X |  |  |  |  |
| Subject Consent Signed | X |  |  |  |  |
| Clinical Assessment | X | X | X | X | X |
| SF-36 and Fatigue Questionnaire |  | X | X | X | X |
| Dispense Study Supplements |  | X | X | X | X |
| Cognitive Behavioral Intervention (weekly) |  | X | X | X | X |
| Record Study Drug Use |  |  | X | X | X |
| Record Adverse Events |  |  | X | X | X |
| End Study Interview |  |  |  |  | X |
| Phone Contact Available | X | X | X | X | X |

#
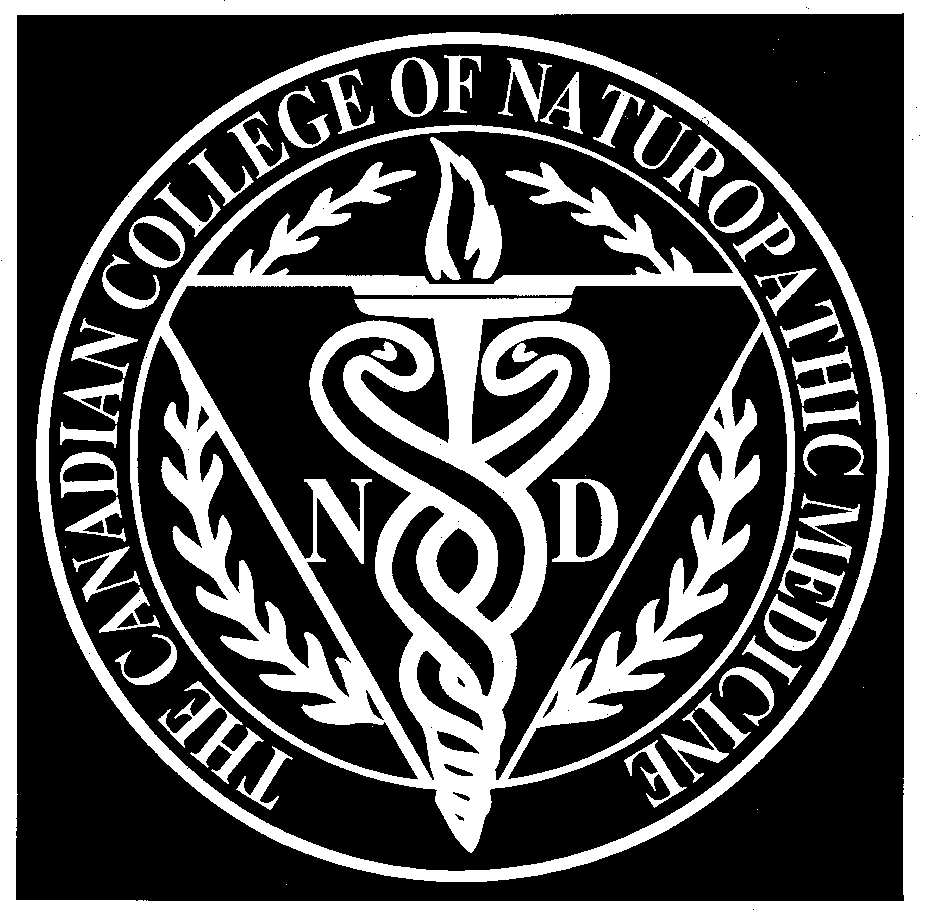
THE CANADIAN COLLEGE OF NATUROPATHIC MEDICINE

**Treatment of anxiety amongst Canada Post workers: A randomized controlled trial**

# Principal investigator: Ed Mills

# Study Co-ordinators: Orest Szczurko and Kieran Cooley

**Study Title:**

Treatment of anxiety amongst Canada Post workers: A randomized controlled trial

By signing and dating this page of the Case Report Form for the study and subject identified above, I declare that informed consent was obtained from the subject, and that the information contained on the attached pages of the subject’s Case Report Forms correspond to this patient. The information herein –

1. Has been reviewed by me or my delegate, and
2. Is accurate, and
3. Includes the results of tests and evaluations performed on the dates specified.

#
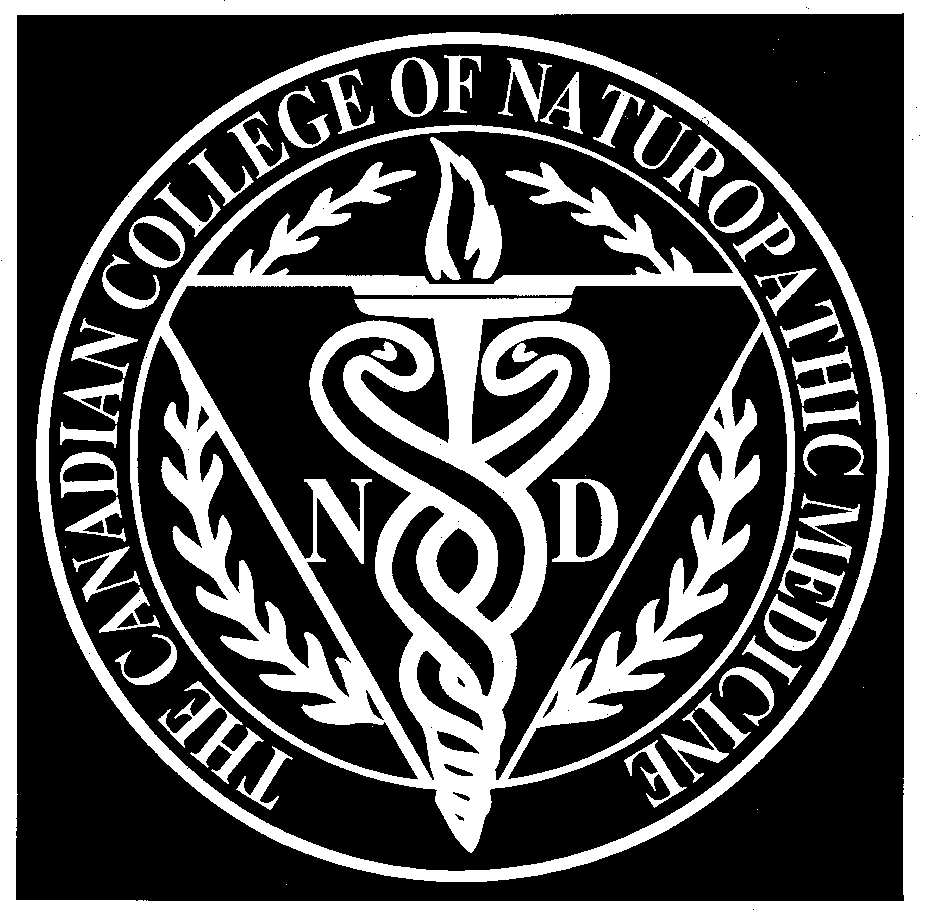
THE CANADIAN COLLEGE OF NATUROPATHIC MEDICINE

**Treatment of anxiety amongst Canada Post workers: A randomized controlled trial**

# Principal investigator: Ed Mills

# Study Co-ordinators: Orest Szczurko and Kieran Cooley

#

# Randomization number assigned to this subject __________

# Study Coordinator: __________________________ date: _____________

#
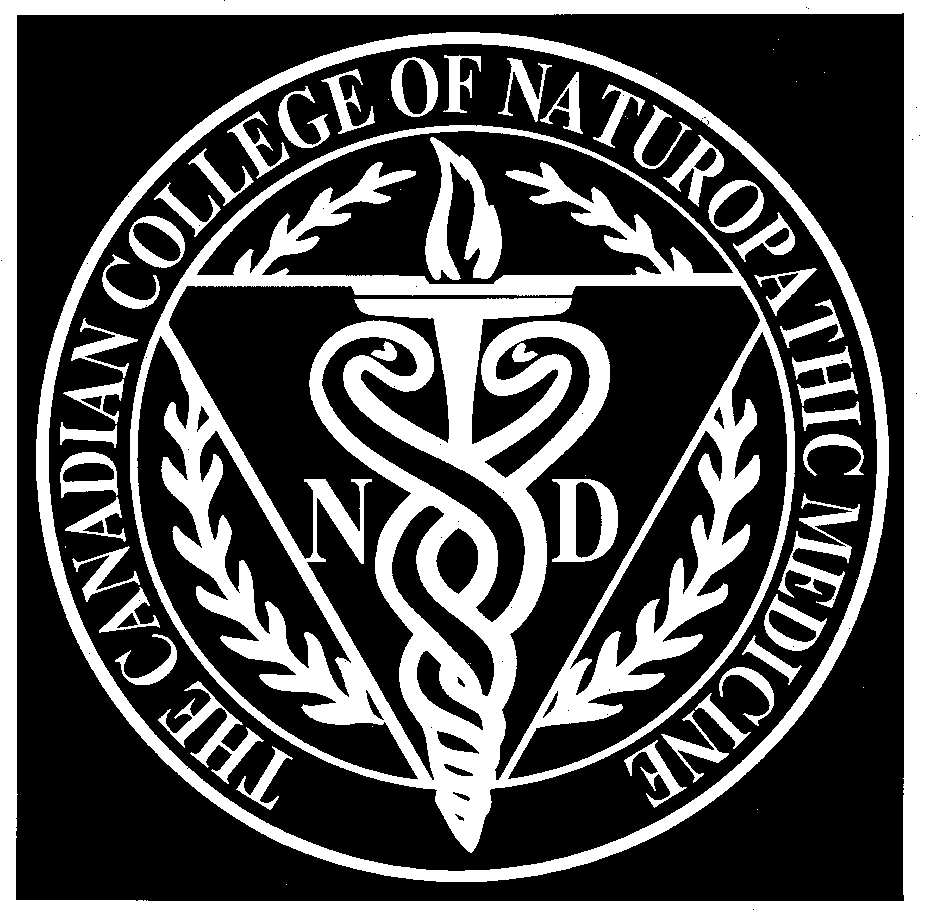
THE CANADIAN COLLEGE OF NATUROPATHIC MEDICINE

**Treatment of anxiety amongst Canada Post workers: A randomized controlled trial**

# Principal investigator: Ed Mills

# Study Co-ordinators: Orest Szczurko and Kieran Cooley

# Subject initials: ___________ Subject number: __________

| **INCLUSION CRITERIA** | Y/N |
| --- | --- |
| 1. Mentally competent subjects who are available for the entire study period and willing to adhere to the protocol requirements and able to give informed consent by signing the informed consent form, which is also signed by a witness. |  |
| 2. Subjects between 18 and 65 years of age, with low back pain for at least six weeks. |  |
| 3. Normal in terms of physical examination at the pre-study screening medical, or in the case of an abnormality, the medical practitioner considers the abnormality to be clinically insignificant. |  |
| 4. Minimum score of 10 on the Beck Anxiety Index |  |
|  |  |
| EXCLUSION CRITERIA | Y/N |
| 1. Any history or other condition which the study physician regards as clinically significant to the study (including allergies or sensitivities to withanolides or multivitamins). |  |
| 2. A major illness considered to be clinically significant by the study physician within 3 months of the study start date. |  |
| 3. Participation in another study. |  |
| 4. Pregnancy or intent to become pregnant in the next 6 months |  |
| 5. Medication (benzodiazepines) that is contraindicated with herb/multi-vitamin |  |
| 6. Alcoholism or substance abuse (sedative) |  |
| 7. Current history of tumors. |  |
| 8. Any other serious conditions |  |

#

# Study Coordinator: __________________________ date: _____________


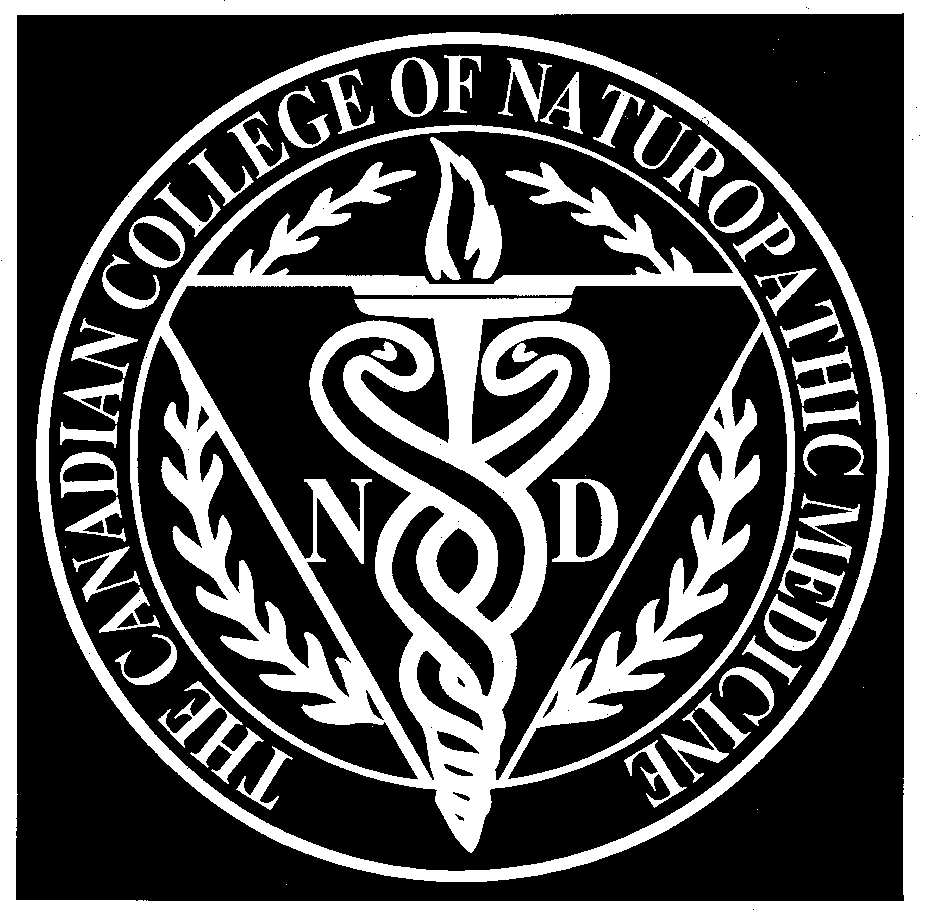

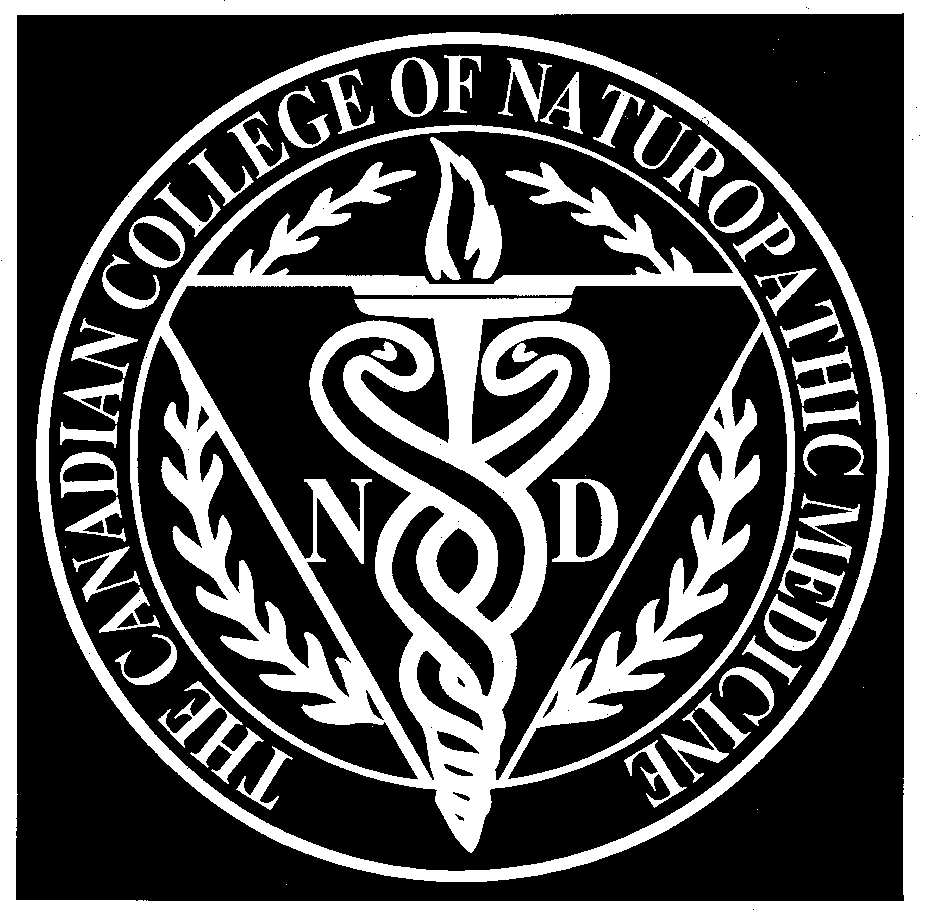
 THE CANADIAN COLLEGE OF NATUROPATHIC MEDICINE

**Treatment of anxiety amongst Canada Post workers: A randomized controlled trial**

# principal investigator: Ed Mills

# Study Co-ordinators: Orest Szczurko and Kieran Cooley

# Demographic information and Medical history

Subject name: _________________________________ Subject number: _____________________

Screening date: ________________________________ Subject initials: ______________________

Date of Birth: _________________________________ Ethnicity: __________________________

Address: _______________________________________________ Post Code_______________

Telephone: (H)____________________ (W)_____________________ (M)______________________

Marital status: ________________________________ Occupation: _____________________________

Does the patient suffer from a disease other than the study specific inclusion criteria? Yes No

**If yes, specify below:**

Asthma Yes No

Hypertension Yes No

Allergy / hay fever Yes No

Endocrine and Metabolism Yes No

Nervous system Yes No

Cardiovascular system Yes No

Respiratory system Yes No

Digestive system Yes No

Musculoskeletal system Yes No

Genitourinary system Yes No

Emotional illness Yes No

Other Yes No

Alcohol intake _______ drinks / day _______drinks / week

Cigarettes consumed _______ cigarettes / day _______ packs / week _______ # of years

Current medication Yes No

**If yes, list below and enter on concomitant form:**

Examining Physician: __________________________ date: _____________


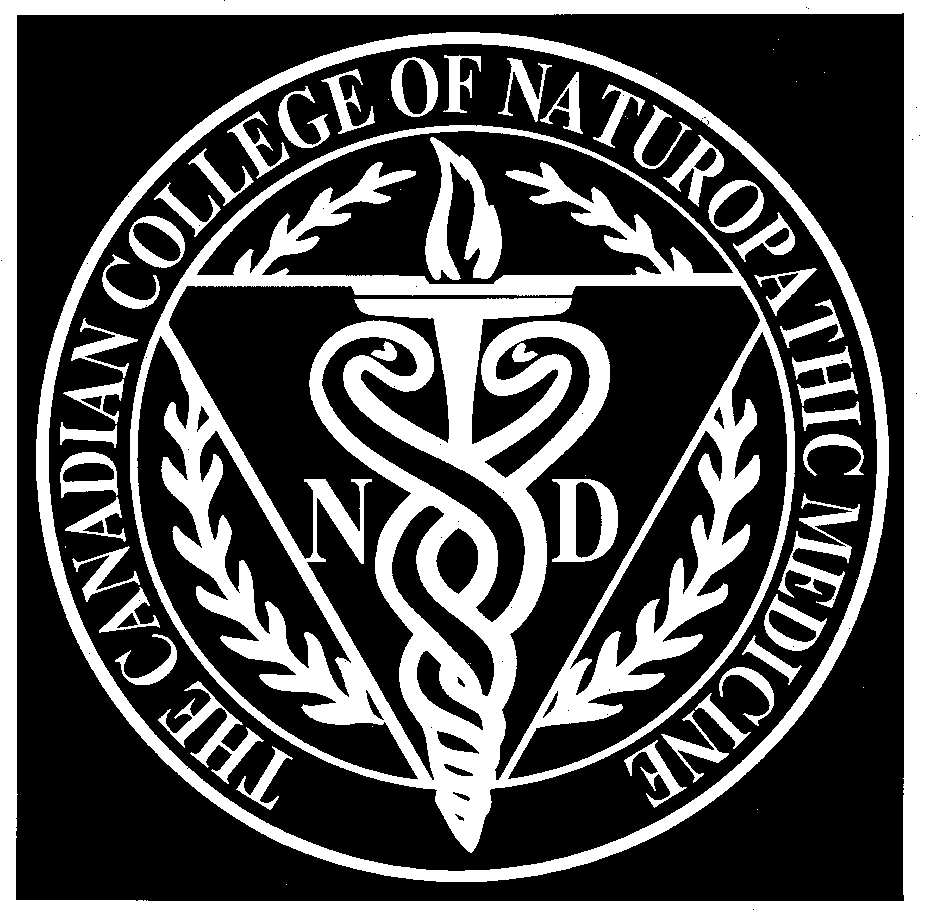
THE CANADIAN COLLEGE OF NATUROPATHIC MEDICINE

**Treatment of anxiety amongst Canada Post workers: A randomized controlled trial**

# Principal investigator: Ed Mills

# Study Co-ordinators: Orest Szczurko and Kieran Cooley

# Baseline measurements

Height: _______________ (m)

Weight: _______________ (kg)

BMI:_________________ (kg/m^2^)

Blood pressure:_______________ (mm hg)

Orthostatic blood pressure: __________________ (mm hg)

Pulse_______________ (bpm)

Temp_______________ (^0^C)

Respiration rate___________ (bpm)

Body Fat______________ (%)

# Examining Physician: __________________________ date: _____________

#
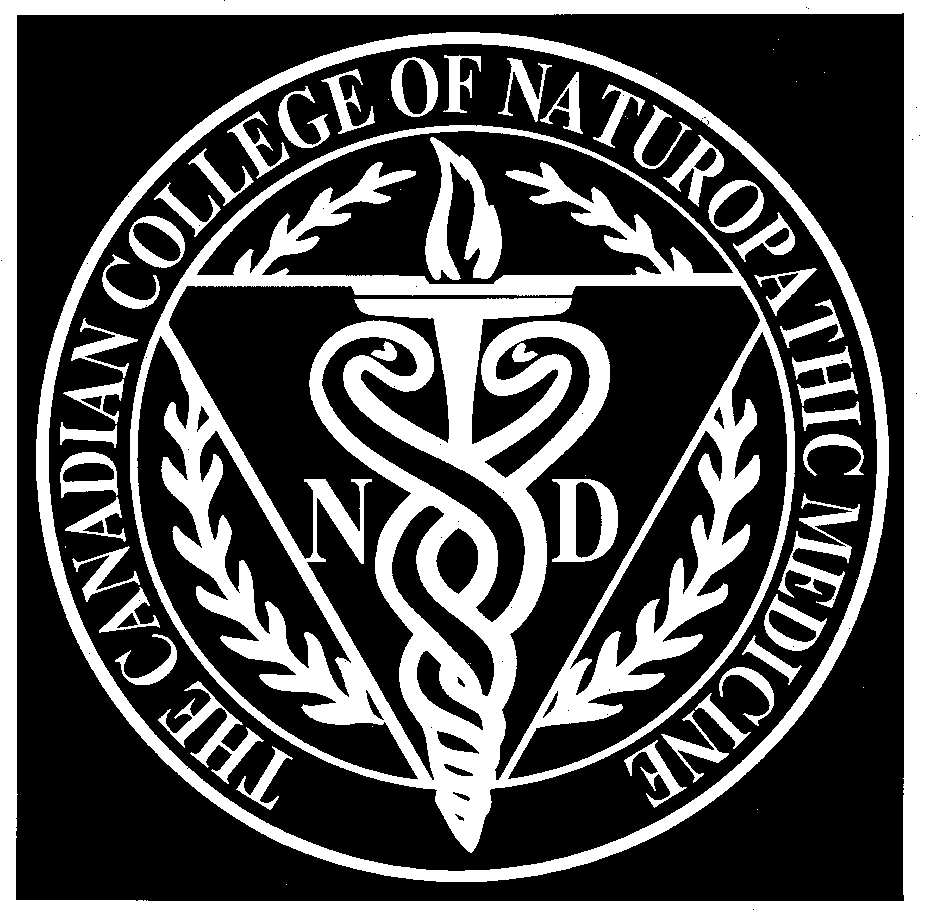
THE CANADIAN COLLEGE OF NATUROPATHIC MEDICINE

Subject name: _______________________________ Screening date: ________________

#

# NOTES: Generic or trade name; Use trade name for fixed combinations only

Route: PO: oral,

IV: intravenous bolus,

INF: intravenous infusion

IM: intra-muscular

O: other

**Other therapy should not, if possible, be changed during the study**

# Examining Physician: __________________________ date: _____________

#
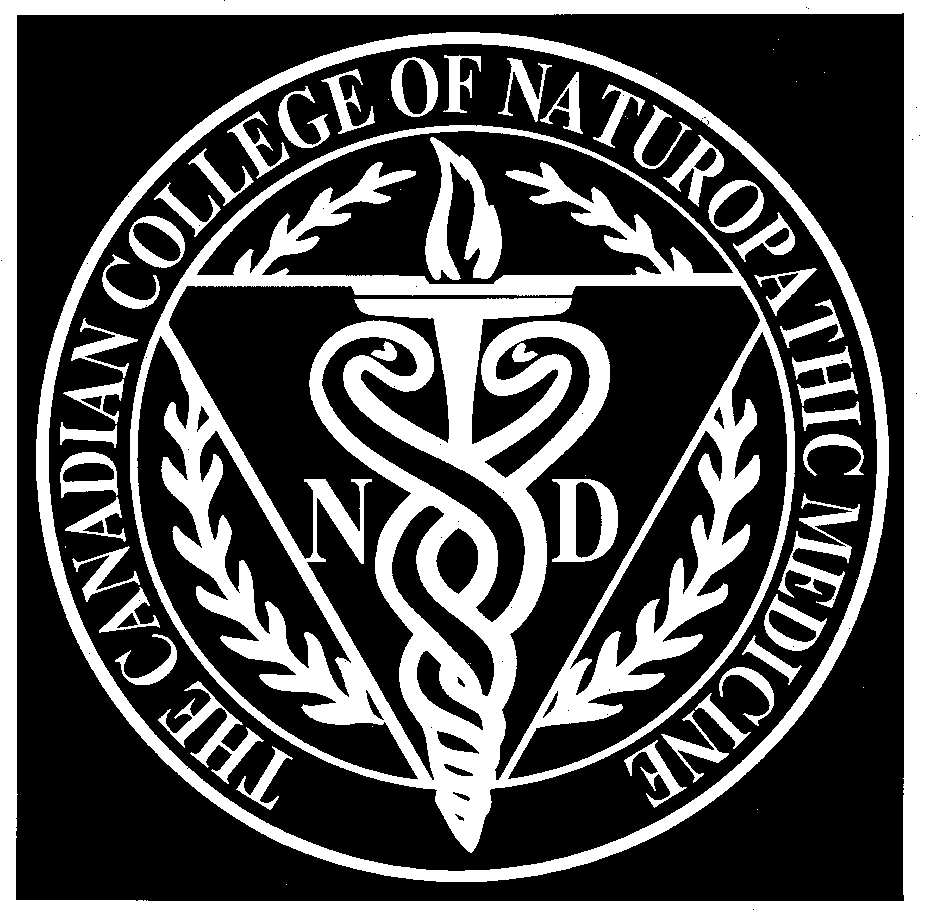
THE CANADIAN COLLEGE OF NATUROPATHIC MEDICINE

Subject initials: ___________________ Subject number:______________________

Day X indicates an extra day if the patient needed to come for assessment. In the case of other days being required, a

Separate form will be written up.

# Examining Physician: __________________________ date: _____________

# Study Coordinator: __________________________ date: _____________

#
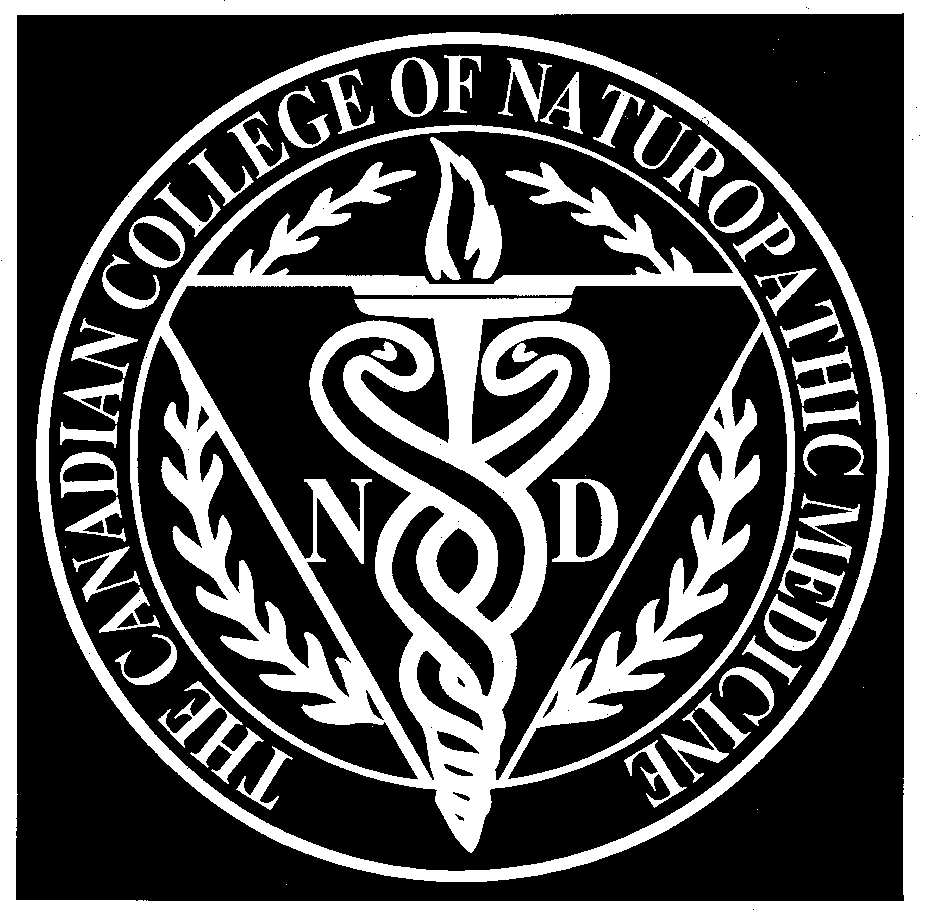
THE CANADIAN COLLEGE OF NATUROPATHIC MEDICINE

Subject initials________________________ Subject number ______________________

Day 0 date ___/___/___ Day 49 date ___/___/___ Day 98 date ___/___/___

# Change to concomitant medication

Day 1 Has therapy changed since last visit? Yes No

*(please specify below)*

Day 28 Has therapy changed since last visit? Yes No

*(please specify below)*

Day 56 Has therapy changed since last visit? Yes No

*(please specify below)*

Day 84 Has therapy changed since last visit? Yes No

*(please specify below)*

*Other day_____* Has therapy changed since last visit? Yes No

*(please specify below)*

# NOTES: Generic or trade name; use trade name for fixed combinations only

Route; PO: oral, IV: intravenous bolus, INF: intravenous infusion, O: other

**Other therapy should not be changed.**

Day 1 Date _______________ Examining Physician:_________________________________________

Day 28 Date _______________ Examining Physician:_________________________________________

Day 56 Date _______________ Examining Physician:_________________________________________

Day 84 Date _______________ Examining Physician:_________________________________________

Other Date _______________ Examining Physician:_________________________________________

#
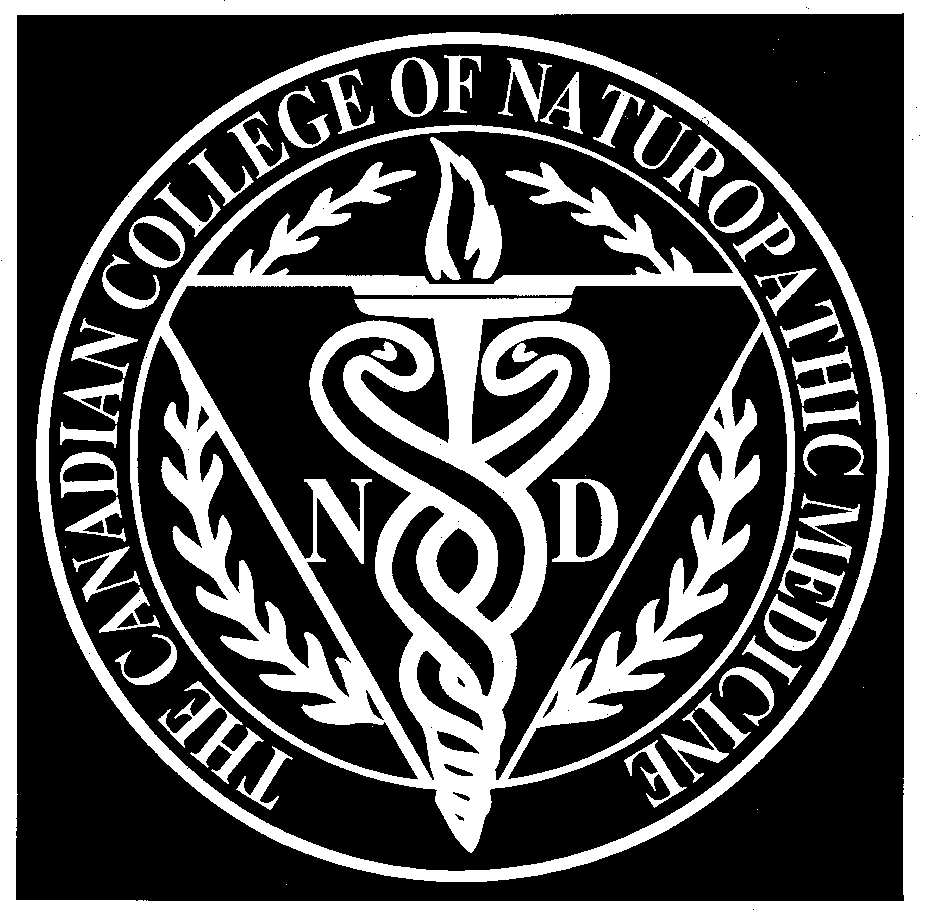
THE CANADIAN COLLEGE OF NATUROPATHIC MEDICINE

**Withdrawal from Study**

Subject initials________________________ Subject number ______________________

Withdrawal date (dd/mm/yy): __/__/__

Was withdrawal associated with an adverse event (AE)? Yes No

Was withdrawal associated with a serious adverse event (SAE)? Yes No

Was withdrawal associated with a trial supplement related AE? Yes No

Was withdrawal associated with a trial supplement related SAE? Yes No

**Reason for withdrawal:**

**Examining Physician: _________________________ date: _____________**

# Study Coordinator: __________________________ date: _____________

#
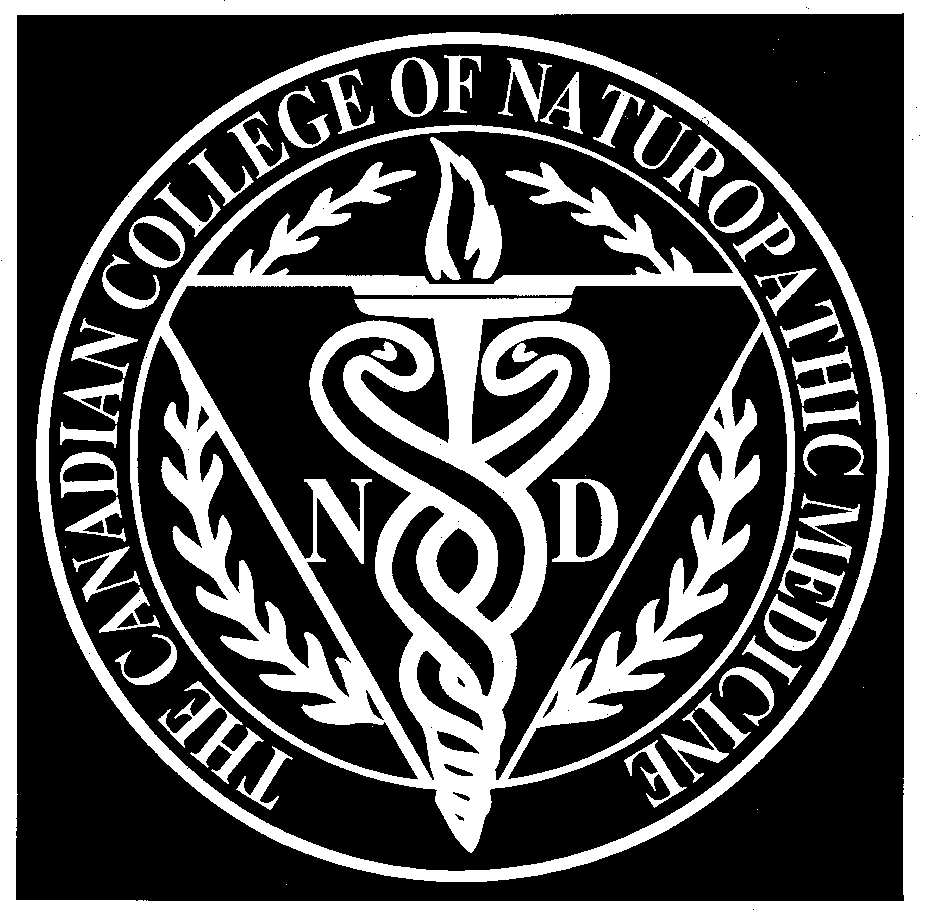
THE CANADIAN COLLEGE OF NATUROPATHIC MEDICINE

**Adverse Event Form**

Subject initials________________________ Subject number ______________________

#

# Examining Physician: _________________________ date: _____________

# Study Coordinator: __________________________ date: _____________

## Appendix E References

1. Merck Manual of Diagnosis and Therapy, 17’th ed. Beers, M; Bertow R. *Chapter 187 – Anxiety Disorders*. Merck & Co, Inc. 2005.

2. Brawman-Mintzer O, Yonkers KA. *New trends in the treatment of anxiety disorders*. CNS Spectr 2004;9(8 Suppl 7):19-27.

3. Howell HB, Brawman-Mintzer O, Monnier J, Yonkers KA. *Generalized anxiety disorder in women*. Psychiatr Clin North Am 2001;24(1):165-78.

4. Lepine JP. The *epidemiology of anxiety disorders: prevalence and societal costs*. *J Clin Psychiatry* 2002;63 Suppl 14:4-8.

5. Marciniak M, Lage MJ, Landbloom RP, Dunayevich E, Bowman L. *Medical and productivity costs of anxiety disorders: case control study.* Depress Anxiety 2004;19(2):112-20.

6. Ferris’ Clinical Advisor.

## 7. Gingrich, P.M. and C.I. Fogel, *Herbal therapy use by perimenopausal women.* J Obstet Gynecol Neonatal Nurs, 2003. 32(2): p. 181-9.

## 8. Ernst, E., *Rise in popularity of complementary and alternative medicine: reasons and consequences for vaccination.* Vaccine, 2001. 20 Suppl 1: p. S90-3; discussion S89.

## 9. Ernst, E., *The Desktop Guide to Complementary and Alternative Medicine.* Harcourt, Toronto, 2001.

## 10. Ernst, E., *The risk-benefit profile of commonly used herbal therapies: Ginkgo, St. John's Wort, Ginseng, Echinacea, Saw Palmetto, and Kava.* Ann Intern Med, 2002. 136(1): p. 42-53.

## 11. Ernst, E., *Serious psychiatric and neurological adverse effects of herbal medicines -- a systematic review.* Acta Psychiatr Scand, 2003. 108(2): p. 83-91.

## 12. Cetaruk, E.W. and C.K. Aaron, *Hazards of nonprescription medications.* Emerg Med Clin North Am, 1994. 12(2): p. 483-510.

13. Bhattacharya SK, Bhattacharya A, Sairam K, Ghosal S. *Anxiolytic-antidepressant activity of Withania somnifera glycowithanolides: an experimental study*. Phytomedicine 2000;7:463-469.

14. Bhattacharya A, Ghosal S, Bhattacharya SK. *Antioxidant effect of Withania somnifera glycowithanolides in chronic footshock stress-induced perturbations of oxidative free radical scavenging enzymes and lipid peroxidation in rat frontal cortex and striatum*. J Ethnopharmacol 2001;74:1-6.

15. Singh B, Saxena AK, Chandan BK, et al. *Adaptogenic activity of a novel, withanolide-free aqueous fraction from the root of Withania somnifera*. Phytother Res 2001;15:311-318.

16. Archana R, Namasivayam A. *Antistressor effect of Withania somnifera*. J Ethnopharmacol 1999;64:91-93.

17. Dhuley JN. *Adaptogenic and cardioprotective action of ashwagandha in rats and frogs*. J Ethnopharmacol 2000;70:57-63.

18. Grunze, H. *Modulation of neural cell memberane conductance by the herbal anxiolytic and antiepileptic drug aswal.* Neuropsychobiology, Vol  42(Suppll), Nov 2000.

19. Rainey JM Jr, et al. *Specificity of lactate infusion as a model of anxiety*. Psychopharmacol Bull 20(1):45-9,1984.

20. Straznicky NE, Louis WJ, McGrade P, Howes LG. *The effects of dietary lipid modivication on blood pressure, cardiovascular reactivity and sympathetic activity in man*. J Hypertens 11(4):427-37, 1993.)

21. Monteiro MG et al. *Subjective feelings of anxiety in young men after ethanol and diazepam infusions*. J Clin Psychiatry 51(1):12-16, 1990.

22. Somer E; Tamir E; Maguen S; Litz BT. *Brief cognitive-behavioral phone-based intervention targeting anxiety about the threat of attack: a pilot study.* Behav Res Ther. 2005 May; Vol. 43 (5), pp. 669-79.)

23. Leahy

24. Rosner B., *Fundamentals of Biostatistics*. 4’th Ed. Section 8, 10. 2001.
